# Identification and validation of a prognostic signature of cuproptosis-related genes for esophageal squamous cell carcinoma

**DOI:** 10.18632/aging.205012

**Published:** 2023-09-02

**Authors:** Yiping Zhang, Kebing Chen, Liyan Wang, Juhui Chen, Zhizhong Lin, Yuanmei Chen, Junqiang Chen, Yu Lin, Yuanji Xu, Haiyan Peng

**Affiliations:** 1 Department of Radiation Oncology, Clinical Oncology School of Fujian Medical University, Fujian Cancer Hospital, Fuzhou 350014, China; 2 The First Clinical Medical College, Xuzhou Medical University, Xuzhou 221004, China; 3 Department of Thoracic Surgery, Clinical Oncology School of Fujian Medical University, Fujian Cancer Hospital, Fuzhou 350014, China; 4 Department of Clinical Laboratory, The School of Clinical Medicine, Fujian Medical University, The First Hospital of Putian, Putian 351199, China

**Keywords:** esophageal squamous cell carcinoma, cuproptosis, prognostic signature, risk score, nomogram

## Abstract

Esophageal squamous cell carcinoma (ESCC) is a highly lethal form of cancer. Cuproptosis is a recently discovered form of regulated cell death. However, its significance in ESCC remains largely unknown. In this study, we observed significant expression differences in most of the 12 cuproptosis-related genes (CRGs) in the TCGA-ESCC dataset, which was validated using GSE20347, GSE38129, and individual ESCC datasets. We were able to divide patients in the TCGA-ESCC cohort into two subgroups based on disease, and found significant differences in survivor outcomes and biological functions between these subgroups. Additionally, we identified 11 prognosis-related genes from the 12 CRGs using LASSO COX regression analysis and constructed a CRGs signature for ESCC. Patients were categorized into high- and low-risk subgroups based on their median risk score, with those in the high-risk subgroup having significantly worse overall survival than those in the low-risk subgroup. The CRGs signature was also highly accurate in predicting prognosis and survival outcomes. Univariate and multivariate Cox regression analyses revealed that 8 of the 11 CRGs were independent prognostic factors for predicting survival in ESCC patients. Furthermore, our nomogram performed well and could serve as a useful tool for predicting prognosis. Finally, our risk model was found to be relevant to the sensitivity of targeted agents and immune infiltration. Functional enrichment analysis demonstrated that the risk model was associated with biological pathways of tumor migration and invasion. In summary, our study may provide a promising prognostic signature based on CRGs and offers potential targets for personalized therapy.

## INTRODUCTION

Esophageal cancer (EC) remains one of the most common malignancies globally with poor therapeutic outcomes, ranking as the sixth highest cause of cancer-related mortality worldwide [1]. Esophageal squamous cell carcinoma (ESCC) is the predominant subtype of EC, accounting for nearly 90% of EC cases in China [2]. Due to the lack of typical clinical symptoms and effective techniques for early diagnosis, ESCC is often diagnosed at a late stage. Currently, the main treatment options for ESCC include surgery, radiotherapy, chemotherapy, and immunotherapy. Despite advances in therapeutic management, the prognosis for ESCC patients remains bleak, with a 5-year survival rate of only 5% in patients with advanced-stage disease [3]. Therefore, the identification of novel prognostic markers and effective personalized therapeutic targets is urgently needed for the early detection and treatment of ESCC.

Cuproptosis is a unique form of cell death that is distinct from apoptosis, necrosis, and ferroptosis, and was first proposed in 2022 [4]. Tsvetkov et al. discovered that copper-induced death occurs through the binding of copper to lipoylated components of the tricarboxylic acid (TCA) cycle, leading to the aggregation of lipoylated proteins and subsequent loss of iron-sulfur cluster proteins. This results in proteotoxic stress and ultimately cell death [4]. Copper is closely related to tumors [5], and copper depletion by the copper chelating agent tetrathiomolybdate reduces metastasis in triple-negative breast cancer and improves patient prognosis [6]. Studies suggest that copper ionophores, such as disulfiram and elesclomol, could be used as cancer therapeutic agents by inducing copper toxicity [7, 8]. Similarly, nanoparticles loaded with disulfiram/Cu2+ could promote the death of esophageal cancer cells [9]. Therefore, understanding the underlying mechanisms and functions of changes in cuproptosis-related genes (CRGs) is vital for identifying new ways to treat ESCC.

Studying CRGs can also be valuable in predicting cancer prognosis and identifying potential therapeutic targets. Zhou et al. constructed a Cuproptosis Activation Scoring (CuAS) model for glioblastoma based on the TCGA, CGGA, and GEO databases and found that samples with high CuAS had worse prognoses than those with low CuAS [10]. The study also identified epiregulin (EREG) as a core oncogene in glioblastoma that affects immunity by influencing PD-L1 expression [10]. Previous research has shown that low levels of ferredoxin1 (FDX1) expression are closely associated with tumor-lymph node metastasis and short survival in renal clear cell carcinoma [11]. Another study found that knocking out the lipoyltransferase 1 (LIPT1) inhibited the proliferation and invasion of liver cancer cells, suggesting that LIPT1 might be a new therapeutic target for liver cancer [12]. Pyruvate dehydrogenase E1 subunit alpha 1 (PDHA1) has been suggested as a prognostic predictive marker of ESCA by Xu et al. using univariate analysis [13]. These studies have revealed the potential value of CRGs in tumors and may provide new directions for further understanding the specific roles and molecular mechanisms of CRGs. Nevertheless, the role of CRGs in ESCC remains largely unknown.

In this study, we aimed to identify important CRGs and tumor subtypes in ESCC. We used LASSO regression to construct prognostic models, and evaluated the accuracy of prognosis and survival analysis using Kaplan–Meier (KM) curves and receiver operating characteristic (ROC) curves. Univariate and multivariate Cox regression analyses were conducted to demonstrate independent prognostic factors for predicting survival in ESCC patients. We further developed a nomogram to predict patient survival at 1-, 2-, and 3-year time points, and used decision curve analysis (DCA) to evaluate the predictive effect of the nomogram model. Additionally, we performed functional enrichment analysis, immune infiltration analysis, and drug sensitivity analysis based on the risk model. These analyses synthesize the role of CRGs in various aspects of ESCC, emphasize the importance of CRGs in the development of ESCC, and provide knowledge for the therapeutic application of a CRGs signature in ESCC.

## MATERIALS AND METHODS

### Data download and arrangement

We used the TCGABiolinks R package [14] to download the ESCC dataset (TCGA-ESCC) from The Cancer Genome Atlas database (TCGA, https://portal.gdc.cancer.gov/) and analyzed it as the test set. We removed samples lacking clinical information data and retained only primary tumor (-01A) tissue types, resulting in a count of sequencing data comprising 80 ESCC samples with clinical information. We normalized sequencing data from the TCGA cohort from raw counts to fragments per kilobase million (FPKM). Corresponding clinical information of patients was obtained from the UCSC Xena database (http://genome.ucsc.edu) [15], and mutation data were taken from MAF files downloaded from the TCGA database. Furthermore, we used a unified standardized TCGA-ESCC dataset, which integrated the TCGA and GTEx databases and the sequencing data in the form of normalized read counts as Transcripts per Million (TPM). The TCGA-ESCC dataset consisted of 745 samples (653 normal samples from GTEx and 92 tumor tissues from TCGA), and was used for subsequent differential expression analysis of CRGs between normal and ESCC groups.

Furthermore, we downloaded two microarray gene-expression profiles, GSE2034 [16] and GSE38129 [17], from the Gene Expression Omnibus (GEO, https://www.ncbi.nlm.nih.gov/geo/) [18] using the GEOquery package [19] for use as the verification cohort. These cohorts were obtained from Homo sapiens and were converted into corresponding genes using the annotation information available from the gene platforms, namely GPL571 (HG-U133A_2) Affymetrix Human Genome U133A 2.0 Array. The GSE20347 cohort included 17 para-carcinoma tissue samples and 17 carcinoma tissue samples, while the GSE38129 cohort included 30 tumor tissue samples and 30 tumor-adjacent tissues. Additionally, we used the gene expression dataset of ESCC patients obtained from our own sequencing for correlation analysis.

The individual ESCC dataset comprised 12 samples, including expression profile data from six esophageal squamous carcinoma tissue samples (grouping: ESCC) and six adjacent normal esophageal tissues (grouping: Normal). These samples were obtained from surgical patients at Fujian Cancer Hospital between July 1st and July 17th, 2020, and none of the patients had received treatment before surgery. The research scheme (including specimen collection) was reviewed and approved by the Biomedical Ethics Committee of Fujian Cancer Hospital (batch number: K2021-027-01). The clinical data for sequencing of patients with esophageal squamous cell carcinoma in our institution was shown in Supplementary Table 1. The GSE20347, GSE38129, and ESCC datasets were used as validation sets for subsequent validation.

We then searched for twelve CRGs from published literature [4] by using “Cuproptosis-related genes” as the search keyword on PubMed. The 12 CRGs were ATPase copper transporting beta (ATP7B), cyclin dependent kinase inhibitor 2A (CDKN2A), dihydrolipoamide S-acetyltransferase (DLAT), dihydrolipoamide dehydrogenase (DLD), FDX1, glutaminase (GLS), lipoic acid synthetase (LIAS), LIPT1, metal regulatory transcription factor 1 (MTF1), PDHA1, pyruvate dehydrogenase E1 (PDHB), and solute carrier family 31 member 1 (SLC31A1).

### Differential expression analysis and consensus clustering analysis for cuproptosis-related genes

First, we compared the expression profiles (HTSeq-TPM) of cuproptosis-related genes (CRGs) between para-carcinoma tissues and carcinoma tissues. Next, we preprocessed somatic mutation data obtained from the TCGA-ESCC cohort using the “VarScan” software and visualized it using the “maftools” R package [20].

To verify the rationality of clustering, we employed the consensus clustering algorithm, which involves multiple iterations over subsamples of the dataset to provide an indication of cluster stability and parameter decisions. We used subsampling to induce sampling variability [21] and classified ESCC into various clusters to explore the function of CRGs. We applied the “ConsensusClusterPlus” R package [22] with 50 iterations, 80% resampling rate Pearson correlation, clusterAlg = “km”, and distance = “Euclidean” (available at http://www.bioconductor.org/). We investigated gene expression arrays in the ESCC groups to compare the group expression difference of CRGs among different disease subtypes from the TCGA-ESCC cohort using the Wilcoxon rank sum test. CRGs with a *P*-value < 0.05 were considered as threshold values for identification.

### Gene set variation analysis

Gene Set Variation Analysis (GSVA) [23] is a non-parametric unsupervised analysis method primarily used to assess the results of gene set enrichment in microarrays and transcriptomes. It evaluates whether different pathways are enriched between samples by converting the gene expression matrix between samples into the expression matrix of gene sets between samples. To further analyze the differences in biological behavior between ESCC disease subtypes, we conducted GSVA analysis. We used the gene set “h.all.v7.4.symbols.gmt” from the Molecular Signature Database (MSigDB) [24] as the reference set for GSVA. A threshold value of *P* < 0.05 was considered significant.

### Single-sample gene-set enrichment analysis and cuproptosis score

The single-sample gene-set enrichment analysis (ssGSEA) algorithm quantifies the relative enrichment of each gene in a given dataset sample. We used the R package GSVA to calculate the cuproptosis score (CPs) using the ssGSEA algorithm based on the expression matrix of 12 CRGs for each sample in the TCGA-ESCC, GSE20347, GSE38129, and ESCC datasets. We then analyzed the expression differences in CPs between the normal subtype and ESCC subtype in the GSE20347 and GSE38129 cohorts, as well as the expression differences in grouping of different subtypes of ESCC disease in the TCGA-ESCC dataset using the Wilcoxon rank sum test. A *P*-value < 0.05 was set as the cutoff for significance.

### Construction of a prognostic risk model based on CRGs

To construct a prognostic signature and screen prognostic CRGs in the TCGA-ESCC dataset, we utilized the glmnet package [25] to perform Least absolute shrinkage and selection operator (LASSO) Cox regression [26]. The LASSO regression is based on linear regression and adds a penalty term (absolute value of lambda × slope) to reduce overfitting and improve the generalization ability of the model. We performed the analysis using CRGs in the TCGA-ESCC dataset with a random seed number of “2021” and tenfold cross-validation. The results were visualized, and we illustrated the grouping of each sample in the LASSO regression prognostic model according to risk score and survival outcome, as well as the molecular expression of prognostic CRGs in each subgroup by risk factor plots.

The ESCA patients were stratified into low-risk and high-risk subgroups based on the median risk score in the TCGA cohort. Kaplan-Meier curves were generated using the “survival” package to compare the overall survival (OS) differences between the low- and high-risk groups. Furthermore, receiver operating characteristic (ROC) curves [27] and corresponding area under the curve (AUC) values were calculated using the “timeROC” package [28] to evaluate the performance of the prognostic risk model for predicting patient outcomes.

### Drug sensitivity analysis

We collected ESCC cell lines and their drug response data from The Genomics of Drug Sensitivity in Cancer (GDSC, https://www.cancerrxgene.org/) database [29]. To compare the difference in immune drug sensitivity between high- and low-risk patients, we used the “oncoPredict” package [30] to perform drug sensitivity analysis in the TCGA-ESCC cohort. Additionally, the “DESeq2” R package [31] was utilized to screen for differentially expressed genes (DEGs) between the high- and low-risk groups in the TCGA-ESCC cohort, with screening criteria of an adjusted *P*-value < 0.05 and | log2 Fold Change (FC)| ≥1.0. Upregulated genes were screened using the criteria of logFC > 1.0 and an adjusted *P*-value < 0.05. Downregulated genes were screened using the criteria of logFC < −1.0 and an adjusted *P*-value < 0.05. We visualized the results of the expression analysis using the “ggplot2” R package.

### Genomic and functional analysis

Gene Ontology (GO) functional analysis [32] is a widely used approach for large-scale functional enrichment studies, encompassing biological process (BP), molecular function (MF), and cellular component (CC) categories. In our study, we employed the “ClusterProfiler” package [33] to conduct GO enrichment analysis of the differentially expressed genes (DEGs) between low- and high-risk groups in TCGA-ESCC cohort. We considered a statistically significant result when the false discovery rate *q*-value was less than 0.10 and the *P*-value was less than 0.05. The Benjamini-Hochberg method (BH) was utilized to correct the *P*-values.

Gene Set Enrichment Analysis (GSEA) [34] is a computational method that determines whether a defined set of genes exhibits statistically significant concordant differences between two biological states. In this study, GSEA was performed using the “clusterProfiler” R package to elucidate significant functional and pathway differences between the high and low expression groups of DEGs in the TCGA-ESCC cohort. Each analysis procedure was repeated 1000 times. The c2.cp.v7.2.symbols set was downloaded from the MSigDB database and used as the reference gene set. A functional or pathway term with an adjusted *P*-value < 0.05 and a false discovery rate (FDR) < 0.25 was considered to have statistically significant enrichment.

### Immune infiltration analysis

CIBERSORTx [35] is a suite of machine learning tools for assessing immune cell types and their abundance from mixed cell populations. In this study, we uploaded the gene expression data to the CIBERSORTx online analytical platform (https://cibersortx.stanford.edu/) to assign values for immune cell infiltration using the LM22 gene signature. We used boxplots to visualize differences in immune cell infiltration between low- and high-risk groups in the TCGA-ESCC cohort. The Spearman algorithm and “ggplot2” package were used to visualize correlations between low- and high-risk groups. The “pheatmap” package in R software was used to visualize correlations of infiltrating immune cells and CRGs among the low- and high-risk groups in the TCGA-ESCC cohort using a heatmap.

### Construction and validation of the prediction nomogram

To define the clinical value of the CRGs prognostic model constructed by LASSO regression for ESCC, we performed univariate Cox regression analysis on the expression of prognostic CRGs in the TCGA-ESCC dataset. We included all factors from the univariate Cox regression analysis in the multivariate Cox regression analysis to construct a multivariate Cox regression model. Based on the results of the multivariate Cox regression analysis, we established a nomogram using the rms R package. A nomogram [36] is a graph that represents the functional relationship among multiple independent variables through a cluster of disjoint lines in a rectangular coordinate system. It is based on the multivariate Cox regression analysis by setting a certain scale to score and characterize the individual variables within the multivariate Cox regression model, and finally calculating the total score to predict the probability of the event occurrence situation. Furthermore, we used decision curve analysis (DCA) to evaluate the accuracy and discrimination of the nomogram. DCA [37] is a simple method to evaluate clinical predictive models. We also used the R package “ggDCA” [38] to draw the DCA curve to assess the effect of the COX regression model.

### Statistical analysis

All statistical analyses and plots were performed using R software (Version 4.1.2). The difference between two groups was compared using Student’s *t*-test or Wilcoxon rank-sum test. The Kruskal-Wallis test was used to compare differences among three or more groups. For categorical variables, Fisher’s exact test and chi-square test were employed to compare differences between two groups. Spearman’s correlation coefficient was used to assess correlation. All *P*-values were two-sided, and *P* < 0.05 was considered statistically significant.

## RESULTS

### Differential expression of cuproptosis-related genes in ESCC dataset

**Figure 1:**
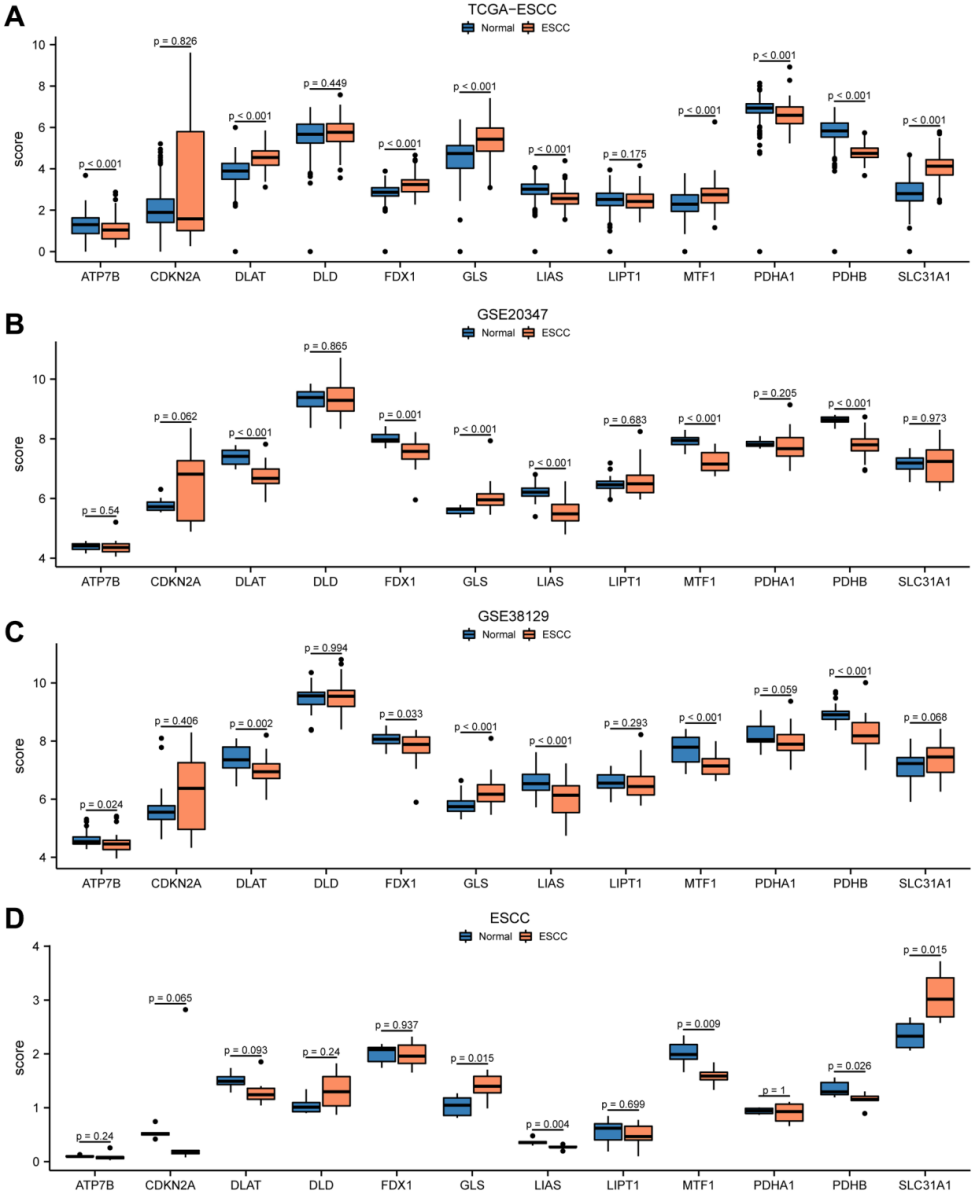
**Differential expression of 12 CRGs between ESCC tissues and normal tissues in four cohorts.** The expression levels of 12 genes from TCGA-ESCC dataset (**A**), GSE20347 dataset (**B**), GSE38129 dataset (**C**), ESCC dataset (**D**) in ESCC tissue and normal tissue.

Firstly, we collected RNA-seq data of 12 CRGs (ATP7B, CDKN2A, DLAT, DLD, FDX1, GLS, LIAS, LIPT1, MTF1, PDHA1, PDHB, SLC31A1) from the TCGA-ESCC dataset, GSE20347 dataset, GSE38129 dataset, and ESCC dataset. We then analyzed the expression differences of these 12 CRGs between ESCC tumor tissues and normal tissues in the four ESCC datasets using the Mann-Whitney *U* test (Wilcoxon rank-sum test). The results of the expression difference correlation analysis were presented through subgroup comparison plots (Figure 1). Our findings revealed that in the TCGA-ESCC dataset, nine out of the 12 CRGs (ATP7B, DLAT, FDX1, GLS, LIAS, MTF1, PDHA1, PDHB, SLC31A1) showed highly statistically significant differences (*P* < 0.001) between the ESCC and normal groups, except for CDKN2A, DLD, and LIPT1 genes (*P* > 0.05) (Figure 1A). In the GSE20347 dataset, the expression of CRGs DLAT, GLS, LIAS, MTF1, and PDHB was highly statistically significant (*P* < 0.001) between the ESCC and normal groups. Additionally, the expression of FDX1 gene was also highly statistically significant (*P* < 0.01) between different subgroups (Figure 1B).

We performed differential expression analysis in the GSE38129 dataset and found that seven out of the 12 CRGs showed statistically significant differences in expression between the ESCC and normal groups, with GLS, LIAS, MTF1, and PDHB genes being highly statistically significant (*P* < 0.001) (Figure 1C). In addition, in the ESCC dataset, the expression of GLS, LIAS, MTF1, PDHB, and SLC31A1 CRGs was statistically significant (*P* < 0.05) between the ESCC and normal groups (Figure 1D). Taken together, these results suggested that the 12 CRGs may play an important role in ESCC based on the differential expression analysis of these genes in the four ESCC datasets.

### Genetic variants of cuproptosis-related genes in ESCC

**Figure 2:**
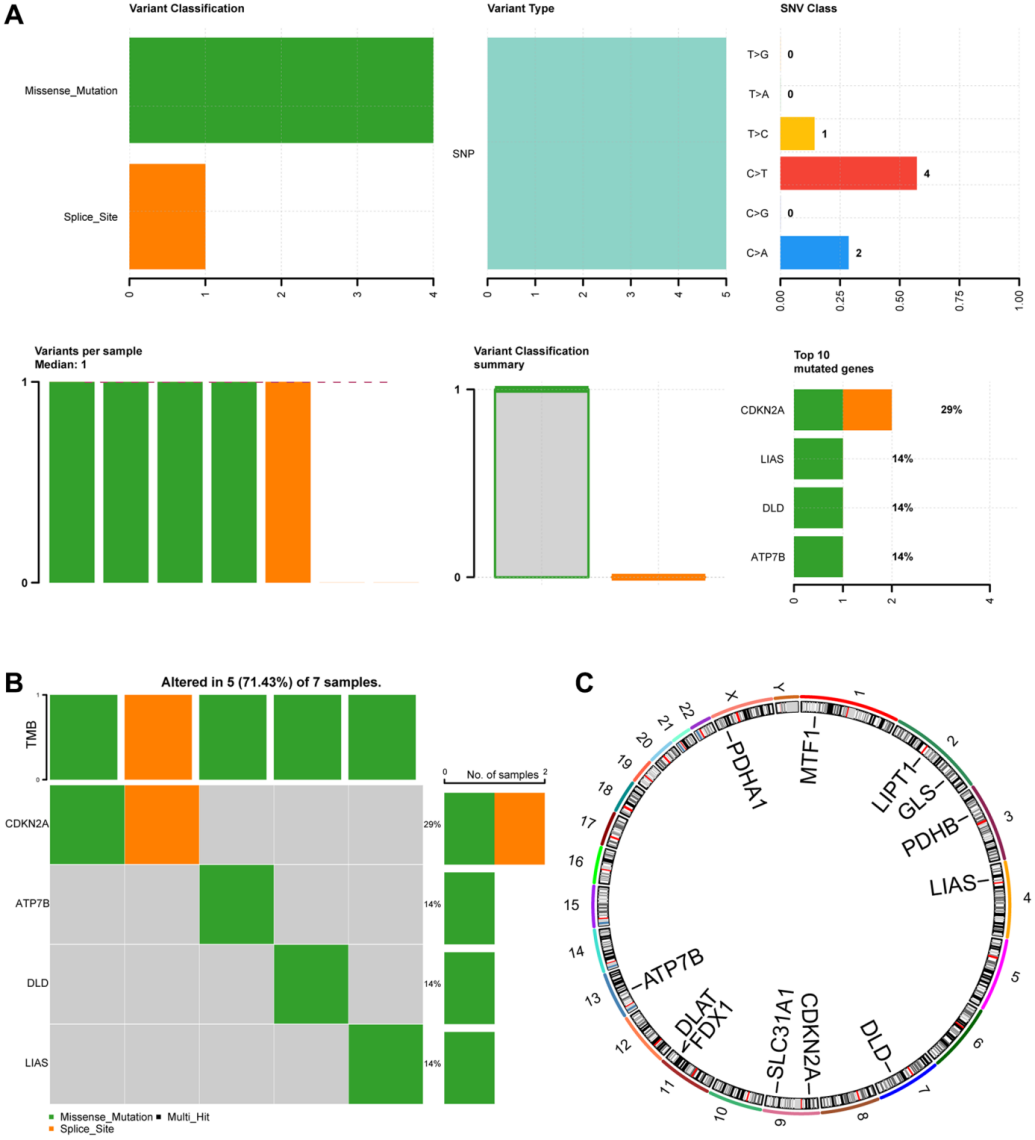
**Mutation analysis of CRGs in ESCC.** (**A**) Demonstration of CRGs mutations in ESCC. (**B**) Mutation details of CRGs are displayed. (**C**) Chromosomal localization map of CRGs.

To investigate somatic mutations in 12 CRGs (ATP7B, CDKN2A, DLAT, DLD, FDX1, GLS, LIAS, LIPT1, MTF1, PDHA1, PDHB, SLC31A1) in ESCC from the TCGA-ESCC cohort, we summarized the results of genetic mutation analysis. Four regulators (CDKN2A, ATP7B, DLD, LIAS) were found to have somatic mutations. The mutations mainly included missense and splice mutations, with missense mutations being the most common. Additionally, all genetic mutations of the 4 CRGs in ESCC were shown to be single nucleotide polymorphisms (SNPs), with C>T being the top-class SNV (Figure 2A).

Figure 2B showed that 7 ESCC samples had somatic mutations, and 5 (71.43%) of the 7 ESCC samples had mutations in the 4 regulators (CDKN2A, ATP7B, DLD, LIAS). CDKN2A mutation samples accounted for 29% of all ESCC genetic mutations, involving missense and splice mutations. The remaining 3 regulator (ATP7B, DLD, LIAS) mutation samples accounted for 14% of all ESCC genetic mutations and only consisted of missense mutations.

Furthermore, we used the RCircos package to map the chromosomal location of the 12 CRGs (Figure 2C). The 12 CRGs were mainly located on chromosomes 1, 2, 4, 9, 11, and X. CRGs were most commonly distributed on chromosomes 2, 9, and 11. Chromosomes 2, 9, and 1 each had 2 CRGs, indicating that CRGs located on the same chromosome might be closely related at the genomic level.

### Development and validation of ESCC subtypes

**Figure 3:**
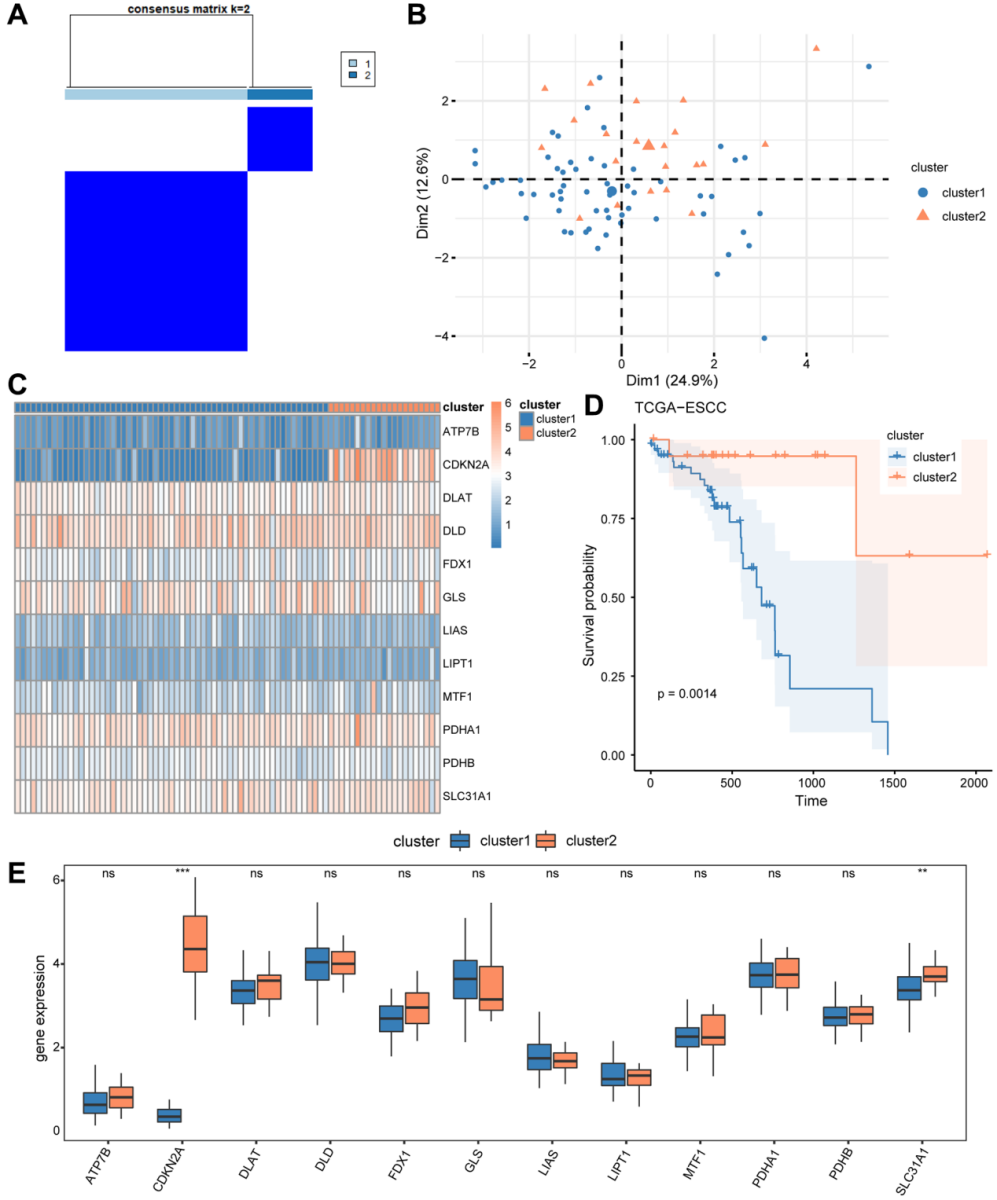
**Construction of disease subtypes associated with ESCC.** (**A**) Results of consensus clustering in ESCC for k = 2 clusters. (**B**) Presentation of PCA results for two ESCC disease subtypes (cluster1 and cluster2). (**C**) Complex numerical heat map of CRGs in different subtypes of ESCC disease. (**D**) KM curve between cluster1 and cluster2. (**E**) The expression level of CRGs in two distinct subtypes. ns: *P* > 0.05, ^*^P < 0.05, ^**^P < 0.01, ^***^P < 0.001.

Initially, we investigated the differential expression of CRGs in ESCC patients from the TCGA-ESCC cohort by using the “ConsensusClusterPlus” R package for consensus clustering analysis based on 12 CRGs (ATP7B, CDKN2A, DLAT, DLD, FDX1, GLS, LIAS, LIPT1, MTF1, PDHA1, PDHB, SLC31A1). This led to the separation of tumor samples into 2 clusters (Figure 3A). Cluster 1 consisted of 59 tumor samples, while cluster 2 contained 21 tumor samples. Next, we performed principal component analysis (PCA) on the expression matrix from these 2 ESCC subtypes. The results showed significant differences in the cuproptosis transcription profiles between cluster 1 and cluster 2 (Figure 3B). We then used a heatmap to identify the gene expression pattern of each subtype (Figure 3C), which revealed a few differences in expression level of the 12 CRGs between the two ESCC subtype from the TCGA-ESCC cohort.

Furthermore, we compared the prognosis signature of these 2 ESCC subtypes using Kaplan-Meier analysis, and the survival analysis showed that cluster 1 had the worst survival situation (Figure 3D). We then used the Wilcoxon rank sum test to compare the level of expression of the 12 CRGs (ATP7B, CDKN2A, DLAT, DLD, FDX1, GLS, LIAS, LIPT1, MTF1, PDHA1, PDHB, SLC31A1) between cluster 1 and 2 from the TCGA-ESCC cohort, and the results are shown in Figure 3E. As a result in Figure 3E, the expression of two regulators (CDKN2A, SLC31A1) showed significant differences between cluster 1 and 2 (*P* < 0.05).

### Gene set variation analysis and construction of cuproptosis score prognosis and diagnostic signature

**Figure 4:**
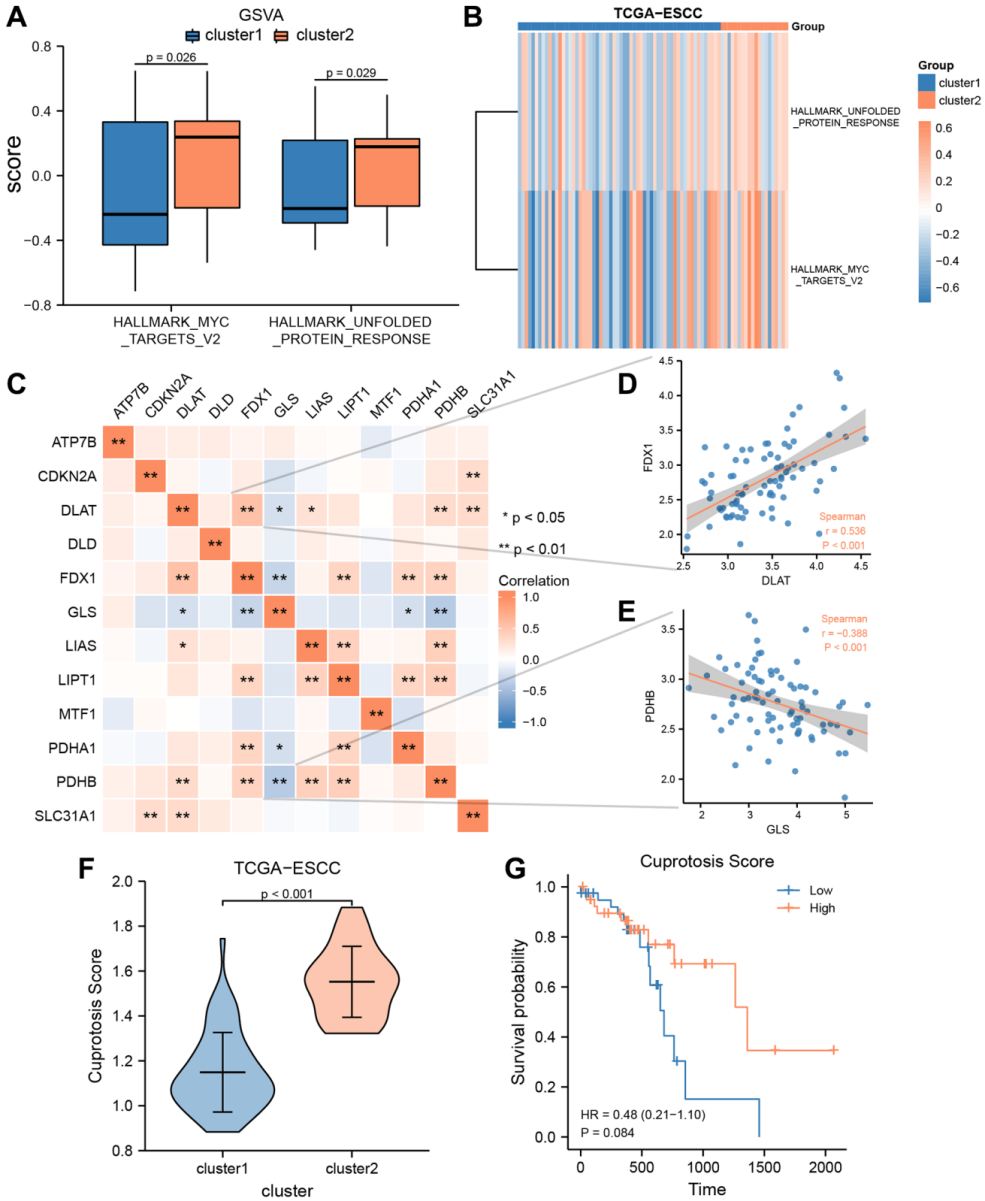
**GSVA and construction of cuproptosis score prognosis signature.** (**A**, **B**) GSVA enrichment analysis between two consensus clusters in TCGA-ESCC dataset, involved group comparison chart (**A**) and ComplexHeatmap (**B**). (**C**) The heat map indicated a correlation among the expression level of 12 CRGs from TCGA-ESCC dataset. (**D**, **E**) Correlation scatter plot showed the correlation between two pairs of CRGs, included DLAT and FDX1 (**D**), GLS and PDHB (**E**). (**F**) Differential analysis of Cuproptosis score among two ESCC subtypes. (**G**) Kaplan–Meier OS curves for patients in the high-and low- score group.

To explore the biological differences between cluster 1 and 2, we conducted GSVA enrichment analysis. Compared to cluster 1, cluster 2 exhibited significantly higher GSVA enrichment scores in two pathways, namely MYC targets V1 and unfolded protein response. The difference in these pathways between the two clusters was further analyzed using Mann-Whitney *U* test and visualized in Figure 4A. Additionally, we used the “pheatmap” R package to create a heatmap to present the specific difference analysis results of these two pathways between cluster 1 and 2, as shown in Figure 4B. The heatmap results confirmed that there were significant differences in these two pathways between cluster 1 and 2 in the TCGA-ESCC cohort.

Spearman correlation analysis was used to investigate the expression levels of 12 CRGs (ATP7B, CDKN2A, DLAT, DLD, FDX1, GLS, LIAS, LIPT1, MTF1, PDHA1, PDHB, and SLC31A1) from the TCGA-ESCC cohort, and the results are presented in Figure 4C. As shown in Figure 4C, only the expression level of GLS was negatively correlated with that of other CRGs, while the expression levels of other CRGs were positively correlated. Additionally, we displayed the correlation scatter plots for the two pairs of genes with the highest and lowest correlation (Figure 4D, 4E). Among the 12 CRGs in the TCGA-ESCC cohort, DLAT and FDX were significantly positively correlated (r = 0.536, *P* < 0.001, Figure 4D), while GLA and PDHB were significantly negatively correlated (r = −0.388, *P* < 0.001, Figure 4E).

Furthermore, to investigate the extent of copper-related cell death in ESCC patients, we utilized the single-sample gene-set enrichment analysis (ssGSEA) algorithm to calculate the Cuproptosis score (CPs) of ESCC patients from the TCGA-ESCC dataset. Subsequently, we used the Wilcoxson rank sum test to examine the difference of CPs between the two subtypes (cluster 1 and 2) in the TCGA-ESCC cohort. We observed a statistically significant difference of CPs between cluster 1 and 2 (*P* < 0.001) (Figure 4F). Then, based on the CRs, we divided the TCGA-ESCC dataset samples into two subtypes (high score group and low score group). Next, we employed KM analysis to investigate the prognostic significance between the two groups using the clinical information of samples from the TCGA-ESCC dataset. As demonstrated in Figure 4G, the CPs might not be able to accurately predict the survival outcome of ESCC patients (*P* > 0.05).

**Figure 5:**
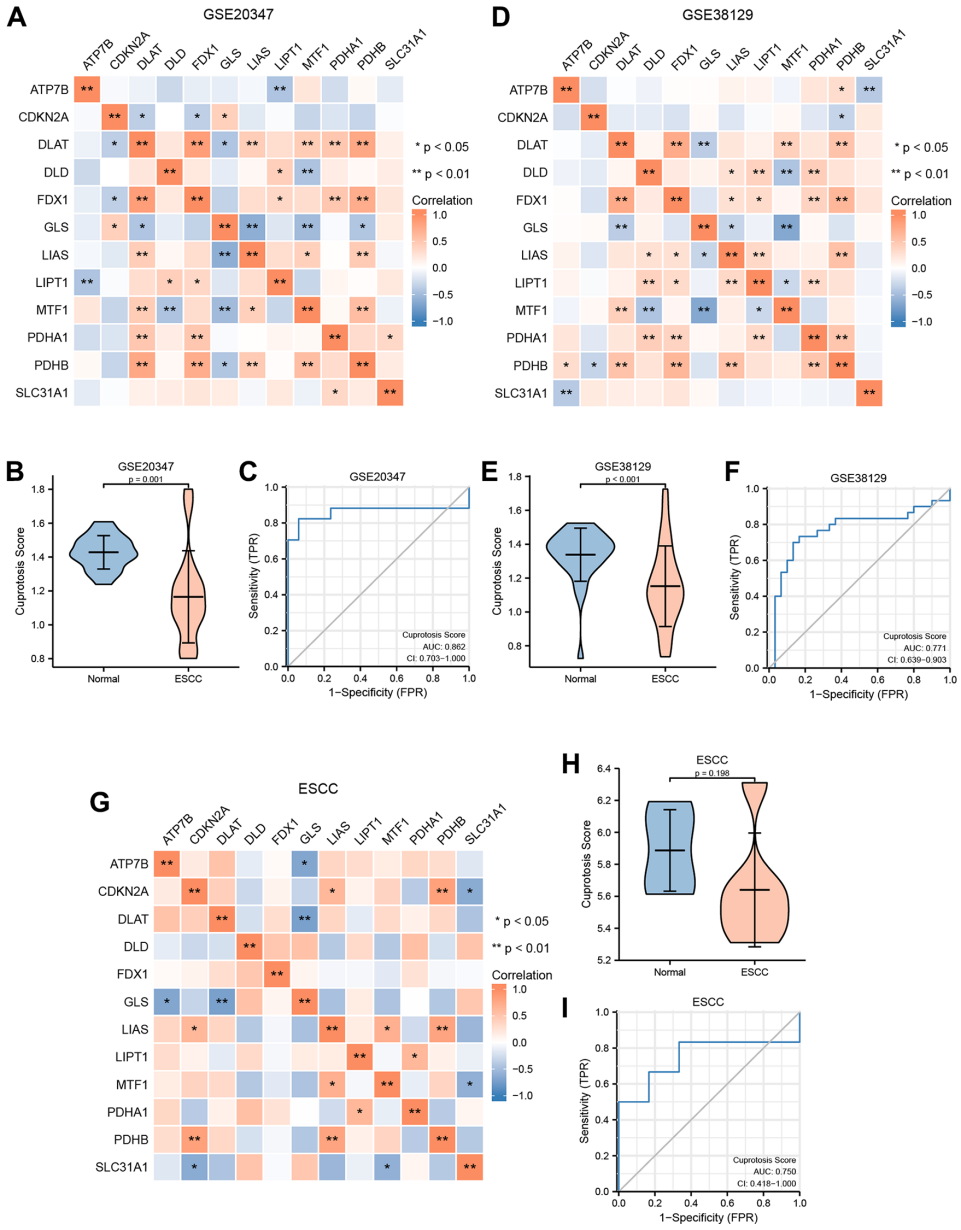
**Construction of cuproptosis score diagnosis signature.** (**A**) The heat map presented the correlation among the expression level of 12 CRGs in GSE20347 cohort. (**B**) Differential analysis of cuproptosis score between normal group and ESCC group in GSE20347 cohort. (**C**) ROC curves showed the diagnosis performance of GSE20347 cohort. (**D**) The heat map presented the correlation among the expression level of 12 CRGs in GSE38129 cohort. (**E**) Differential analysis of cuproptosis score between normal group and ESCC group in GSE38129 cohort. (**F**) ROC curves showed the diagnosis performance of GSE20347 cohort (**C**), GSE38129 cohort. (**G**) The heat map presented the correlation among the expression level of 12 CRGs in ESCC cohort. (**H**) Differential analysis of cuproptosis score between normal group and ESCC group in ESCC cohort. (**I**) ROC curves showed the diagnosis performance of ESCC cohort.

In addition, based on the transcriptome expression matrix of 12 CRGs from three verification datasets (GSE20347, GSE38129, and ESCC), we calculated the correlation expression values among the 12 CRGs using Pearson correlation analysis, and presented the results in heatmaps (Figure 5A, 5D, 5G). According to these heatmaps, we found that the GLS values were negatively correlated with the majority of the other CRGs values in these three verification datasets, while the other CRGs were positively correlated with one another.

Moreover, based on the expression level of the 12 CRGs from these three verification datasets, we utilized the ssGSEA algorithm to evaluate the CPs of each sample. Then, we used the Wilcoxon rank sum test to quantify the difference of CPs between the normal group and the ESCC group in the three verification datasets. The correlation analysis revealed a statistically significant difference of CPs between the normal group and the ESCC group in GSE20347 and GSE38129 (Figure 5B, 5E). Conversely, we found that the difference of CPs was not statistically significant between different groups in the ESCC dataset (Figure 5H).

To explore the clinical utility of CPs evaluation in these three datasets, we utilized a ROC curve to demonstrate its ability to discriminate ESCC diagnosis. As indicated by the ROC curves, the AUC values in the GSE20347 cohort, GSE38129 cohort, and ESCC cohort were 0.862 (Figure 5C), 0.771 (Figure 5F), and 0.750 (Figure 5I), respectively. It can be inferred that the CPs has moderate accuracy in diagnosing ESCC in these three verification datasets.

### Construction and prognostic risk model based on 11 cuproptosis-related genes

**Figure 6:**
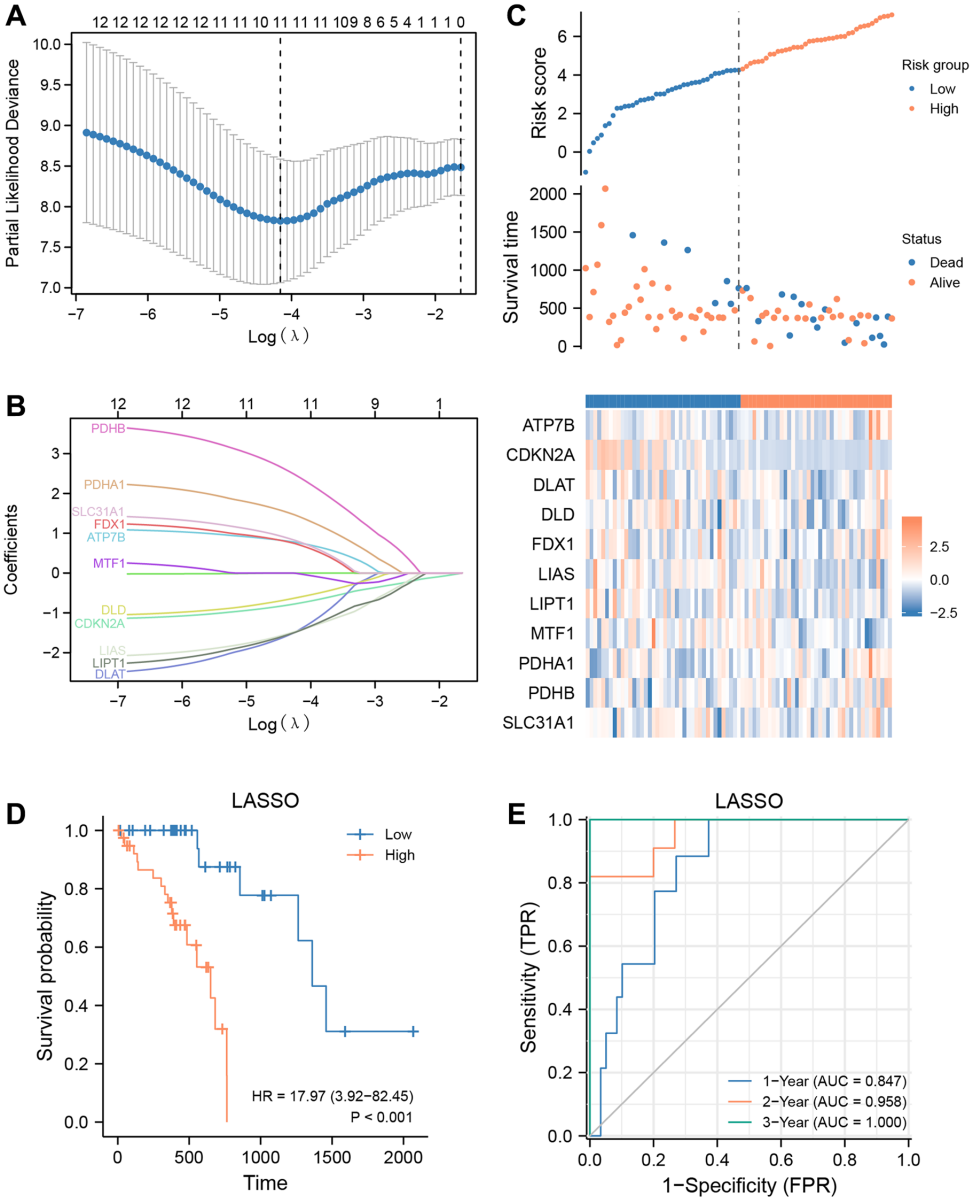
**Prognostic signatures construction and prediction.** (**A**) Partial likelihood deviance of different numbers of variables. One thousand-fold cross-validation was applied for tuning penalty parameter selection. (**B**) LASSO analysis identified 11 CRGs. Each curve corresponds to one gene. (**C**) Risk score, distribution of patient survival status between the low- and high−risk groups, and expression heatmaps of 11 CRGs. (**D**) Kaplan–Meier curves indicated that there is a strong relationship between high and low risk score and the overall survival rate. (**E**) ROC curve was applied to assess the predictive efficiency of the prognostic risk signature.

To evaluate the predictive value of 12 CRGs (ATP7B, CDKN2A, DLAT, DLD, FDX1, GLS, LIAS, LIPT1, MTF1, PDHA1, PDHB, SLC31A1) for clinical outcomes in the TCGA-ESCC dataset, we used the transcriptome expression matrix of 79 patients with ESCC who had a survival time greater than zero to construct a prognosis signature through the LASSO algorithm (Figure 6A, 6B). Supplementary Table 2 shows the signature coefficients for each gene under different penalty coefficients in the LASSO regression. The LASSO regression analysis, which is based on linear regression, removes overfitting and improves generalization ability by increasing the penalty term (lambda times the absolute value of the slope). Based on the LASSO regression analysis, we used 11 CRGs, including ATP7B, CDKN2A, DLAT, DLD, FDX1, LIAS, LIPT1, MTF1, PDHA1, PDHB, and SLC31A1, according to the minimum-minimum criterion and optimal-minimum criterion. We also calculated the penalty coefficients of the CRGs using LASSO analysis and established a risk index by multiplying the gene expression by the corresponding coefficients. We further visualized the sample groupings in the constructed prognostic model for CRGs using risk factor plots (Figure 6C). Kaplan-Meier survival analysis revealed that overall survival was significantly lower in the high-risk group of ESCA patients than in the low-risk group (*P* < 0.05, Figure 6D). The AUC values demonstrated that the risk scores significantly predicted the prognosis of patients with ESCA. As shown in Figure 6E, the AUC values for 1-, 2-, and 3-year were 0.847, 0.958, and 1.000, respectively.

### Evaluation of the sensitivity of antitumor drugs using the signature

**Figure 7:**
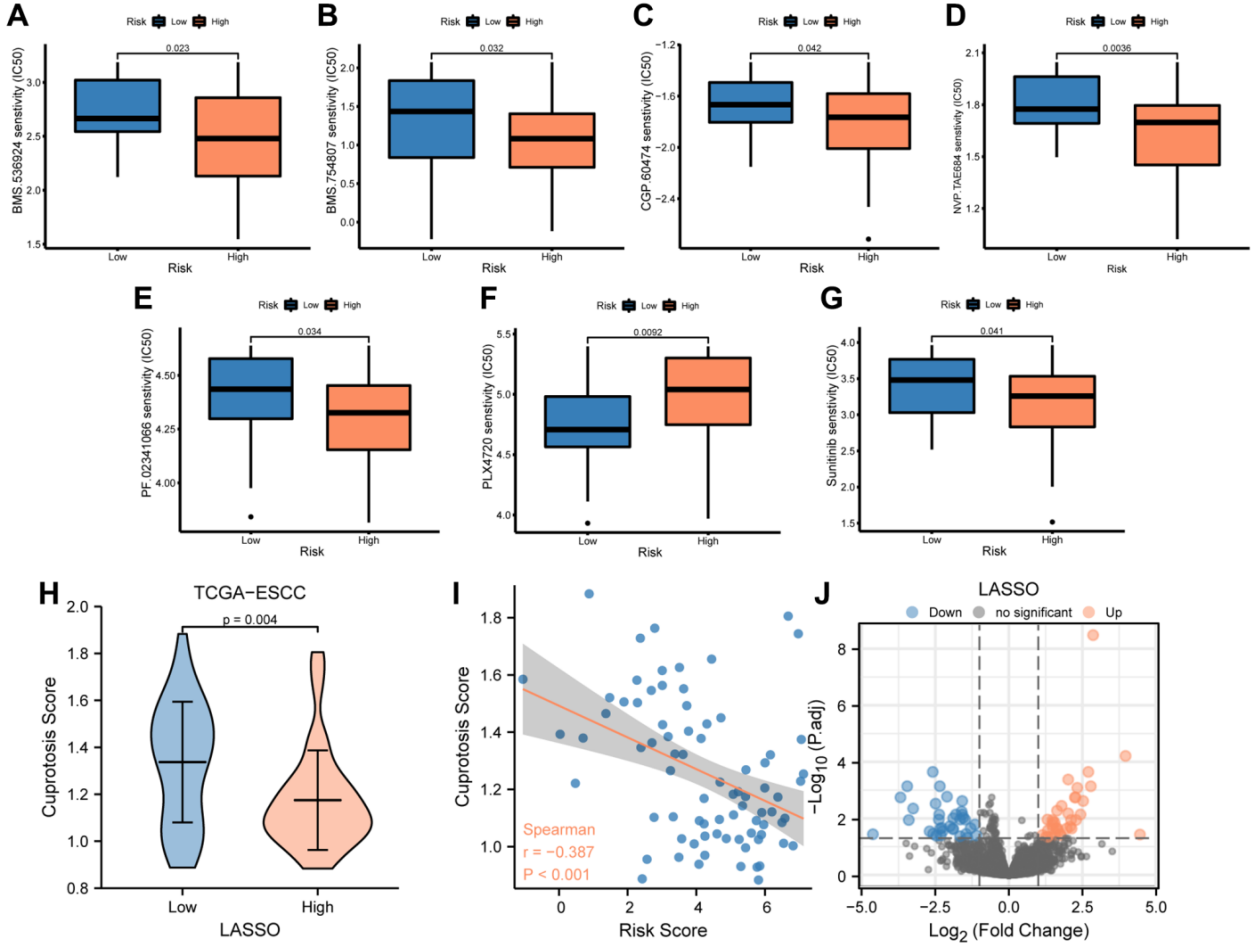
**Analysis of drug sensitivity between low-and high-risk groups.** (**A**–**G**) IC50 of seven drugs, including BMS.536924 (**A**), BMS.754807 (**B**), CGP.60474 (**C**), NVP.TAE684 (**D**), PF.02341066 (**E**), PLX4720 (**F**), and Sunitinib (**G**) differed for ESCC patients in different risk groups. (**H**) Difference in cuproptosis score between low- and high-risk groups in TCGA-ESCC dataset. (**I**) Correlations between cuproptosis score and risk score. (**J**) The plot showed 36 upregulated and 35 downregulated genes based on the above volcano analysis in high-risk group.

The TCGA-ESCC cohort was stratified into low- and high-risk groups using the median risk score. To investigate the differences in drug sensitivity between these groups, we compared the IC50 values of multiple antitumor drugs in both low- and high-risk groups (Figure 7A–7G). Our drug sensitivity analysis revealed significant differences between the low- and high-risk groups in seven drugs, including BMS.536924 (Figure 7A), BMS.754807 (Figure 7B), CGP.60474 (Figure 7C), NVP.TAE684 (Figure 7D), PF.02341066 (Figure 7E), PLX4720 (Figure 7F), and Sunitinib (Figure 7G). Notably, Sunitinib (Figure 7G, *P* < 0.05) was more effective in patients in the low-risk group, whereas the other six drugs were more effective in patients in the high-risk group.

Furthermore, we compared the cuproptosis score (CPs) between the low- and high-risk groups using the Mann-Whitney *U* test and found a significant difference between the two groups (*P* < 0.01, Figure 7H). We then performed a correlation analysis between the risk score and cuproptosis score in patients from the TCGA-ESCC dataset, and the results were visualized by a correlation scatterplot (Figure 7I). Our findings showed that the risk score was negatively correlated with the cuproptosis score in patients from the TCGA-ESCC dataset (r = −0.387, *P* < 0.001, Figure 7I).

To identify the differentially expressed genes (DEGs) associated with risk score in these patients from the TCGA-ESCC dataset, we compared the transcription matrix expression levels in the low-risk and high-risk groups using the DESeq2 package. After analyzing the transcriptomic changes, a total of 18,057 DEGs were identified in the volcano plot (Figure 7J) (Supplementary Table 3). We further screened 71 critical genes (|log (FC)| >1 and FDR <0.05) using R software. Among these, we found 36 upregulated (downregulated in the low-risk group, log (FC) >0) and 35 downregulated (upregulated in the low-risk group, log(FC) <0) transcription factors in the high-risk group.

### Gene ontology (GO) and gene set enrichment analysis (GSEA)

**Figure 8:**
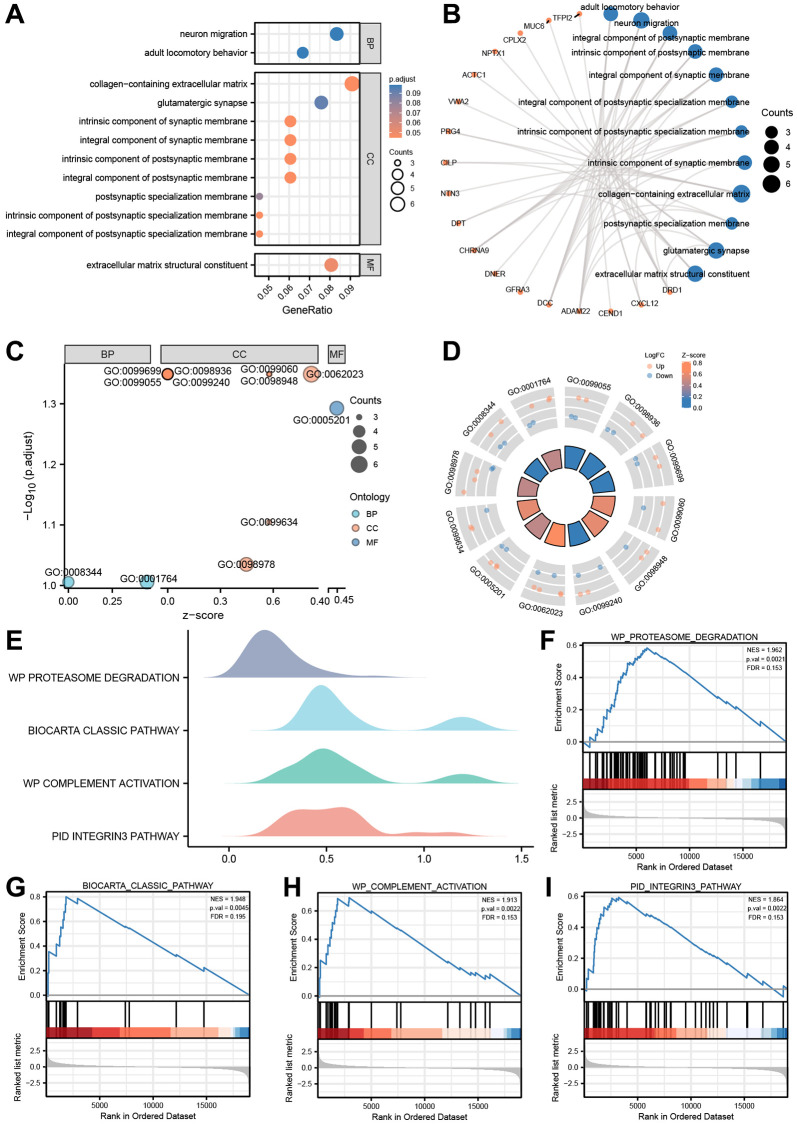
**GO enrichment and genome enrichment analysis.** (**A**) The bubble plot and (**B**) circos plot showing the significantly enriched GO pathways for DEGs between in TCGA-ESCC dataset. (**C**) The bubble plot and (**D**) circle plot presenting the results of GO functional enrichment analysis which standardized by logFC values. (**E**) Four biological characteristics for Gene sets enriched analysis in TCGA-ESCC dataset. (**F**–**I**) The GSEA showed DEGs of TCGA-ESCC dataset significantly enriched in 4 pathways, including the proteasome degradation pathway (**F**), biocarta classic pathway (**G**), complement activation pathway (**H**), and integrin 3 pathway (**I**). Ordinate in bubble plot (**A**) is GO terms, the color of the bubble corresponds to the magnitude of the correlation. In network plots (**B**), orange color dots represented the detail genes, and Navy blue circles represented the detail pathways. In the bubble plot (**C**), Cyan dots represented BP pathway, orange circles represented CC pathway, and the Navy blue circles represented MF pathway. In the circle plot, orange dots represented upregulated genes (logFC > 0), Navy blue dots represented downregulated genes (logFC < 0).

After being converted into gene IDs, the 71 differentially expressed genes (DEGs) were analyzed using Gene Ontology (GO) (Supplementary Table 4). The GO annotations of the DEGs consisted of three parts: CC (cellular component), BP (biological process), and MF (molecular function), which were used to analyze the functional enrichment of the DEGs. The results were visualized in a bubble plot (Figure 8A). The main biological processes involved adult locomotory behavior and neuron migration, while the most abundant cellular component terms were the integral component of postsynaptic membrane, intrinsic component of postsynaptic membrane, integral component of synaptic membrane, and integral component of postsynaptic specialization membrane. The most abundant molecular function terms were extracellular matrix structural constituent. Additionally, we also visualized the results of GO functional enrichment analysis with a circos plot (Figure 8B).

Subsequently, based on the functional enrichment analysis, we calculated the z-score of each gene of the DEGs by providing logFC values obtained from the difference analysis of TCGA-ESCC dataset. The results of GO functional enrichment analysis, which were standardized by logFC values, were presented in a bubble plot (Figure 8C), mainly enriched in cellular component (CC) pathway. The relationship between these pathways and genes was shown in Figure 8D.

We further performed Gene Set Enrichment Analysis (GSEA) on the expression of the DEGs in TCGA-ESCC dataset to uncover signaling pathways that are differently active in ESCC. The GSEA showed that the DEGs of TCGA-ESCC dataset were significantly enriched in four pathways (Figure 8E–8I, Supplementary Table 5, *P* < 0.05 and *q*-value < 0.25), including the proteasome degradation pathway (Figure 8F), Biocarta classic pathway (Figure 8G), complement activation pathway (Figure 8H), and integrin 3 pathway (Figure 8I).

### Characterization of immune infiltration between the low- and high-risk groups

**Figure 9:**
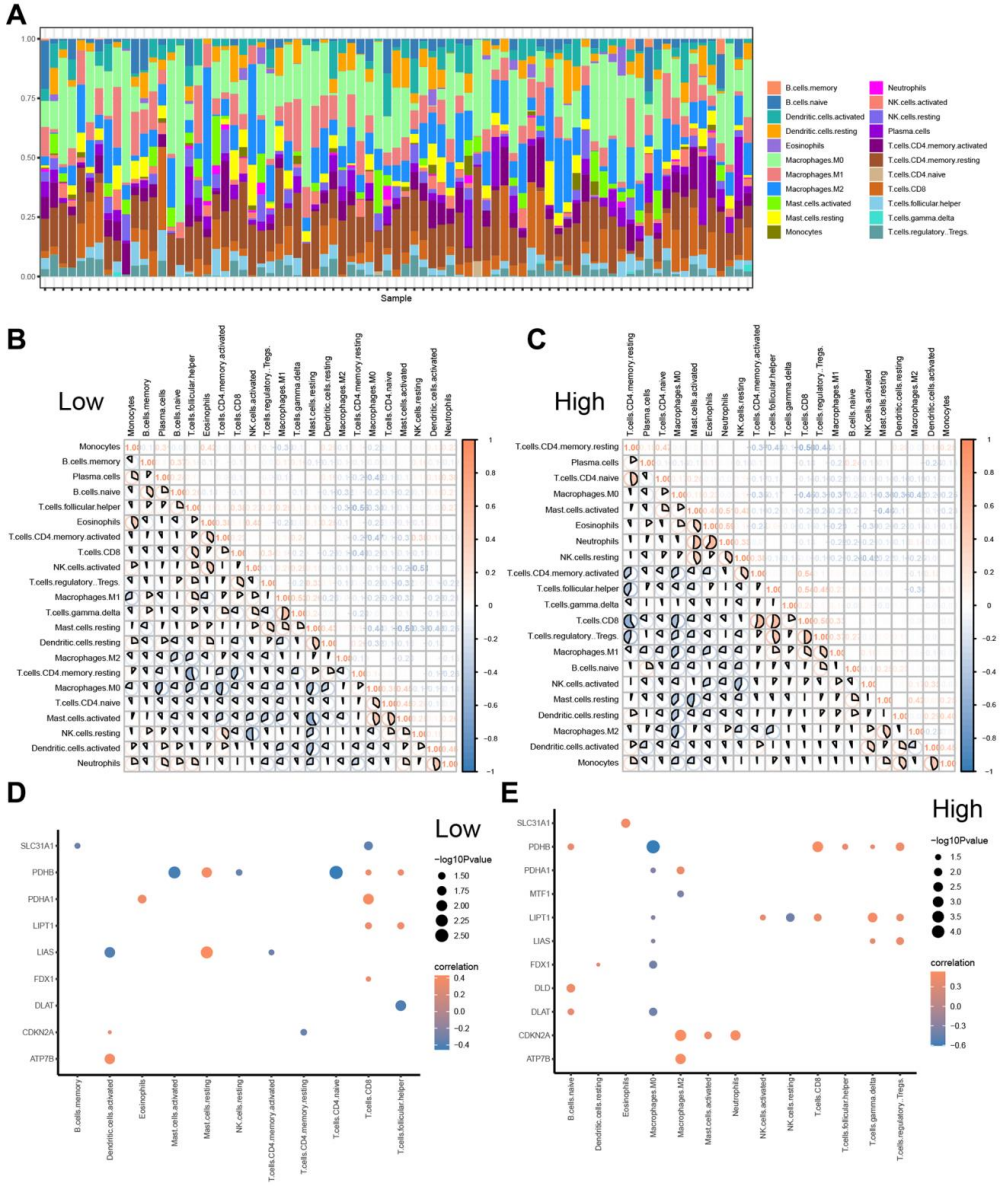
**CIBERSORTX for immune cell infiltration analysis between the low-and high-risk groups.** (**A**) Boxplot present the infiltration abundances analysis of immune cells from TCGA-ESCC cohort by CIBERSORT algorithm. (**B**, **C**) Correlation analysis among infiltration abundance of immune cells in low-risk group (**B**) and high-risk group (**C**) from TCGA-ESCC cohort. (**D**, **E**) Correlation analysis between infiltration abundance of immune cells and expression levels of CRGs in low-risk group (**D**) and high-risk group (**E**).

To explore differences in immune cell infiltration between the low- and high-risk groups, we used CIBERSORT to evaluate the abundance of 22 immune cell types in the TCGA-ESCC cohort. The distribution of overall immune cell abundances among the samples from the TCGA-ESCC cohort is shown in Figure 9A, and the results indicate that Macrophages M0 and Macrophages M1, as well as T cells CD4 memory activated, are the main infiltrating immune cells.

Furthermore, we examined the relationships among the infiltration abundances of the 22 distinct types of immune cell (Monocytes, B cells memory, Plasma cells, B cells naive, T cells follicular helper, Eosinophils, T cells CD4 memory activated, T cells CD8, NK cells activated, T cells regulatory Tregs, Macrophages M1, T cells gamma delta, Mast cells resting, Dendritic cells resting, Macrophages M2, T cells CD4 memory resting, Macrophages M0, T cells CD4 naive, Mast cells activated, NK cells resting, Dendritic cells activated, and Neutrophils) based on the samples with abundances greater than 0 in the low- and high-risk groups (Figure 9B, 9C). Negative correlations were found between most of the 22 types of immune cells in the low-risk group (Figure 9B), while negative correlations were observed between most of the 21 types of immune cells, except B cells memory, in the high-risk group **(**Figure 9C).

Additionally, we investigated the correlation between the expression of 11 CRGs (ATP7B, CDKN2A, DLAT, DLD, FDX1, LIAS, LIPT, MTF1, PDHA1, PDHB, and SLC31A1) and the infiltration abundance of 22 types of immune cells in the low-risk (Figure 9D) and high-risk groups (Figure 9E), respectively. The screening criterion for correlation analysis was a *P* value < 0.05. Our results showed that CRG expression levels were statistically positively correlated with the infiltration abundance of Mast cells resting in the low-risk group, while the infiltration abundances of Mast cells activated and T cells CD4 naive were positively correlated with PDHB expression levels in the low-risk group (Figure 9D). Conversely, most immune cell infiltration abundances were positively correlated with the expression levels of 12 CRGs in the high-risk group (Figure 9E).

### Validation of the prognostic value of the signature

**Figure 10:**
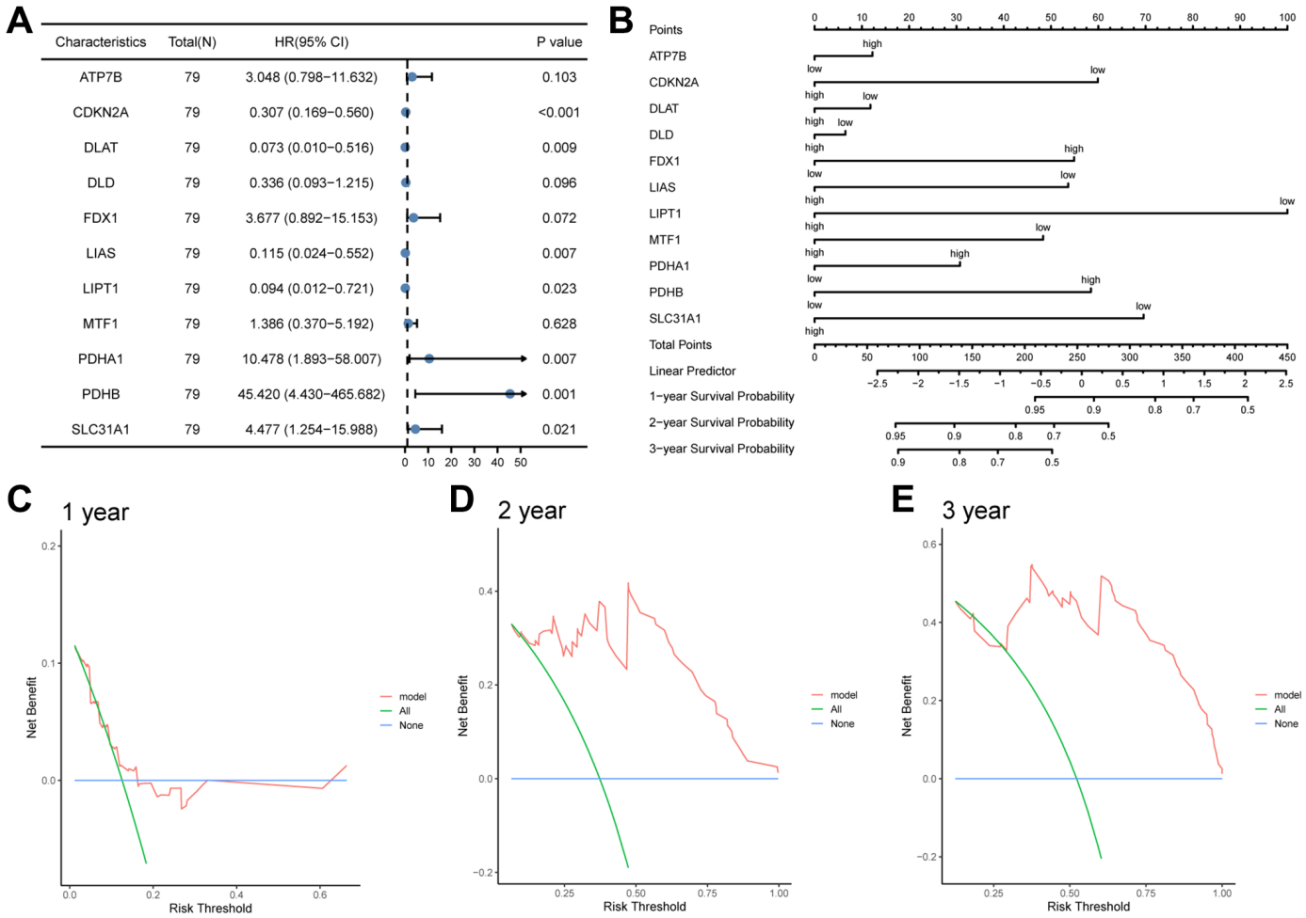
**The prognostic value of the CRGs prognosis model.** (**A**, **B**) Univariate and multivariate cox regression analysis Forest plots (**A**), nomogram (**B**). (**C**, **D**) Decision curve analyses (DCA) of LASSO-Cox regression prognosis model for predicting 1-year (**C**), 2-year (**D**), and 3-year (**E**).

To further validate the prognostic risk model of CRGs, we conducted a statistical analysis of clinical data from ESCC patients obtained from the TCGA-ESCC dataset (Supplementary Table 6). Firstly, we performed univariate and multivariate Cox regression analyses to demonstrate the correlation between patients’ prognosis and the expression levels of 11 CRGs (ATP7B, CDKN2A, DLAT, DLD, FDX1, LIAS, LIPT1, MTF1, PDHA1, PDHB, SLC31A1). In this study, we first conducted univariate Cox regression analysis based on the expression levels of 11 CRGs, followed by multivariate Cox regression analysis including all variables in the univariate Cox analysis (Table 1). Our results revealed that 8 CRGs, namely CDKN2A, DLAT, FDX1, LIAS, LIPT1, PDHA1, PDHB, and SLC31A1, were significantly correlated with ESCC survival (*P* < 0.05). The results of the univariate and multivariate Cox regression analyses were then presented in a forest plot (Figure 10A).

**Table 1:** Cox regression to identify clinical features associated with overall survival (OS).

**Characteristics**	**Total (*N*)**	**Univariate analysis**		**Multivariate analysis**	
		**Hazard ratio (95% CI)**	***P* value**	**Hazard ratio (95% CI)**	***P* value**
ATP7B	79	1.667 (0.528−5.269)	0.384	3.048 (0.798−11.632)	0.103
CDKN2A	79	0.685 (0.516−0.908)	0.009	0.307 (0.169−0.560)	<0.001
DLAT	79	0.663 (0.282−1.559)	0.346	0.073 (0.010−0.516)	0.009
DLD	79	0.667 (0.281−1.585)	0.359	0.336 (0.093−1.215)	0.096
FDX1	79	1.039 (0.457−2.365)	0.927	3.677 (0.892−15.153)	0.072
LIAS	79	0.383 (0.114−1.286)	0.120	0.115 (0.024−0.552)	0.007
LIPT1	79	0.528 (0.202−1.375)	0.191	0.094 (0.012−0.721)	0.023
MTF1	79	0.562 (0.237−1.334)	0.191	1.386 (0.370−5.192)	0.628
PDHA1	79	2.413 (0.854−6.814)	0.096	10.478 (1.893−58.007)	0.007
PDHB	79	2.307 (0.695−7.656)	0.172	45.420 (4.430−465.682)	0.001
SLC31A1	79	1.021 (0.345−3.019)	0.970	4.477 (1.254−15.988)	0.021

Subsequently, based on the statistically significant prognostic factors identified in the multivariate analysis, a nomogram prognostic evaluation model was constructed. A nomogram characterizes multiple variables in the multivariate regression model, and the total score is calculated to predict the probability of events. As shown in Figure 10B, unlike other CRGs, the expression levels of three CRGs, including ATP7B, DLAT, and DLD, exhibited limited usefulness in the Cox regression model.

Afterwards, a Decision Curve Analysis (DCA) was performed to evaluate the clinical value of the LASSO-Cox regression prognosis model for 1-year (Figure 10C), 2-year (Figure 10D), and 3-year (Figure 10E) time points. In the DCA curve, the x-axis represents the threshold probability, while the y-axis displays the net benefit. The results were determined by observing the x-value range of the model line that is higher than all positive line and all negative line. The larger the range of X-value, the better the clinical judgment utility. The DCA analysis revealed that the risk score model constructed by 11 CRGs (ATP7B, CDKN2A, DLAT, DLD, FDX1, LIAS, LIPT1, MTF1, PDHA1, PDHB, SLC31A1) had the best clinical judgment utility for 3-year followed by 2-year and 1-year.

**Figure 11:**
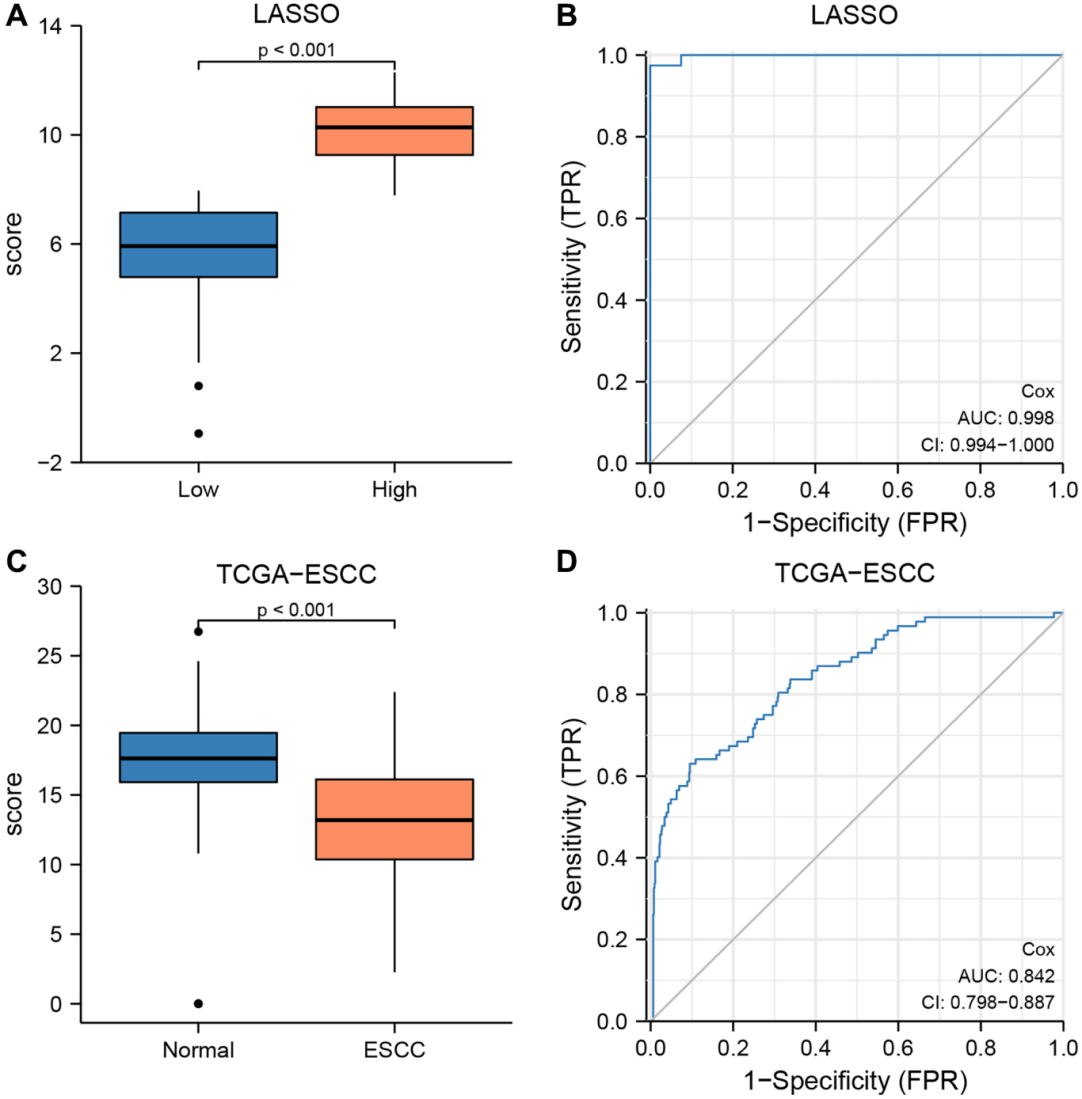
**The prognostic value of the LASSO-Cox regression prognosis risk model in TCGA-ESCC dataset.** (**A**, **B**) Boxplots (**A**) and ROC curve (**B**) for the risk score levels in the low- and high-risk groups in TCGA-ESCC dataset. (**C**, **D**) Boxplots (**C**) and ROC curve (**D**) for the risk score levels in the ESCC and normal groups in TCGA-ESCC dataset.

We verified the diagnostic performance of our risk model using the TCGA-ESCC dataset. Firstly, we calculated the risk score for each sample in the low- and high-risk groups based on the LASSO-Cox regression prognosis model. We then used Mann-Whitney *U* tests to compare the risk scores between the two groups, and found a significant difference (*P* < 0.001, Figure 11A). Subsequently, we generated an ROC curve for the CRG prediction model to evaluate the ability of the risk score system to predict outcomes for low- and high-risk patients (Figure 11B). We observed that the risk score levels exhibited high diagnostic accuracy for grouping patients into low- and high-risk categories (AUC = 0.998, Figure 11B).

Based on the results of multivariate Cox regression analysis, we identified and included 11 CRGs in the construction of the LASSO-Cox regression prognosis model. The formula was as follows:

RiskScores = ATP7B × 1.114 + CDKN2A × −1.180 + DLAT × −2.618 + DLD × −1.091 + FDX1 × 1.302 + LIAS × −2.163 + LTPT1 × −2.366 + MTF1 × 0.326 + PDHA2 × 2.349 + PDHB × 3.816 + SLC31A1 × 1.499.

To validate the diagnostic accuracy of the LASSO-Cox regression prognosis model, we utilized Mann-Whitney *U* tests to calculate the difference in risk scores between the ESCC and normal groups in the TCGA-ESCC cohort (Figure 11C, 11D). Our analysis demonstrated a highly significant difference in risk score between the two groups (*P* < 0.001, Figure 11C), indicating the model’s effectiveness in distinguishing between the two groups. Additionally, we generated an ROC curve to evaluate the diagnostic capacity of the risk score system, with the resulting analysis shown in Figure 11D. The area under the curve (AUC) for the TCGA-ESCC prognostic model was 0.842.

Furthermore, based on the expression levels of 11 CRGs across three cohorts (GSE20347, GSE38129, and ESCC), we calculated the risk score for each sample within the ESCC and normal groups using the formula. We then used Mann-Whitney *U* tests to compare the risk score between ESCC and normal groups in each of the three datasets. Our results showed no statistically significant difference in risk score between the two groups in any of the datasets, including GSE2034 (Supplementary Figure 1A), GSE38129 (Supplementary Figure 1C), and ESCC (Supplementary Figure 1E) (*P* > 0.05). Finally, ROC curve analysis was conducted, and the resulting AUC values for the prognosis model score in GSE2034 (Supplementary Figure 1B), GSE38129 (Supplementary Figure 1D), and ESCC (Supplementary Figure 1F) were 0.623, 0.577, and 0.694, respectively.

## DISCUSSION

In the present study, we identified general differences and positive correlations in the expression of most of the 12 CRGs in the TCGA-ESCC dataset, which were validated by GSE20347, GSE38129, and additional ESCC datasets. Furthermore, the TCGA-ESCC cohort was stratified into two disease subtypes with significant differences in CRG expression, clinical parameters, survival status, and pathway enrichment related to apoptosis regulation. Prognostic scores (high vs. low risk) were established based on eleven prognosis-related genes, which were screened from the 12 CRGs using LASSO COX regression analysis. These patterns effectively predicted the prognosis of ESCC patients. Initial analysis of biological functions in the high-risk versus low-risk groups was performed by GSEA and GO analyses. Moreover, there were differences in prognostic characteristics and drug sensitivity between high and low-risk groups. Additionally, somatic mutations were prevalent in ESCC patients, mostly concentrated in missense mutations, and the SNP rate was high in ESCC. Finally, Univariate and multivariate Cox regression analyses found eight out of 11 CRGs were clinically significantly associated with prognosis. A nomogram was constructed based on the results of multivariate regression analyses, which showed the clinical predictive effect of the LASSO-Cox regression prognostic model with a prediction timeline of 3 years > 2 years > 1 year. This study highlighted the important role of CRGs as prognostic molecular biomarkers in ESCC and provided new ideas for identifying reliable molecular biomarkers to target ESCC therapy.

Initially, we performed differential expression analysis of 12 CRGs (ATP7B, CDKN2A, DLAT, DLD, FDX1, GLS, LIAS, LIPT1, MTF1, PDHA1, PDHB, SLC31A1) in four datasets (TCGA-ESCC, GSE20347, GSE38129, and ESCC datasets). Our results suggested that these 12 CRGs might play a crucial role in ESCC. Notably, we found that GLS expression was significantly higher in ESCC tissues than in para-carcinoma tissues in all four datasets. GLS has been shown to have multiple roles in cancer cells, including maintaining mitochondrial metabolism, activating cell signaling, and promoting cancer cell growth [39]. Previous studies have also implicated GLS as a downstream factor of the proto-oncogene transcription factor c-Jun in the development of breast cancer, as well as in the proliferation of hypoxic gastric cancer cells [40, 41]. Additionally, a study showed that the circ_0001093/miR-579-3p/GLS regulatory network might affect the progression of ESCC [42]. Taken together, these findings suggest that GLS may be a key regulator of prognosis and a potential therapeutic target for ESCC patients.

Based on the transcriptome data of the 12 CRGs, we performed consensus clustering analysis to classify ESCC patients in the TCGA-ESCC cohort into two distinct disease subtypes, namely cluster 1 and cluster 2. We found that patients in cluster 2 had a significantly better prognosis than those in cluster 1. Furthermore, the patients in cluster 2 had higher expression levels of CDKN2A and SLC31A1 and showed significant enrichment of the MYC targets V1 and unfolded protein response pathways. On one hand, CDKN2A is a critical tumor suppressor that induces cell cycle arrest by inhibiting the CDK4/6-Cyclin D complex upon activation [43]. On the other hand, SLC31A1 plays a vital role in maintaining intracellular copper homeostasis [44]. Previous research suggested that CDKN2A and SLC31A1 may be key regulators affecting the prognosis of ESCC patients. Dysregulation of mitochondrial respiration due to intracellular copper overload can lead to cell death, while the activation of the “MYC targets V2” and “unfolded protein response” pathways can promote cell death by regulating apoptosis and cell cycle [45, 46]. Our findings were consistent with previous reports and provided a plausible explanation for the superior prognosis of cluster 2 over cluster 1.

After calculating the cuproptosis scores (CPs) using ssGSEA for each ESCC patient in the TCGA-ESCC dataset, we observed that the CPs were significantly higher in cluster 2 than in cluster 1. However, CPs were not found to be effective in predicting survival outcomes in ESCC patients. In addition, we found that CPs levels were significantly higher in the ESCC group than in the normal group, based on the GSE20347 and GSE38129 datasets. Furthermore, ROC curves indicated that CPs levels could be used for subgroup diagnosis between the normal and ESCC groups, suggesting that CPs levels are a useful diagnostic model for subgrouping.

We then conducted bioinformatics analyses to identify differentially expressed genes (DEGs) between low and high-risk groups. A total of 71 DEGs were identified in the TCGA-ESCC cohort. Gene ontology (GO) analysis revealed that the DEGs were primarily enriched in neuronal migration, synaptic membrane composition, and extracellular matrix (ECM) structure. The ECM is a key component of the tumor microenvironment that promotes tumor proliferation, migration, and invasion [47]. Moreover, gene set enrichment analysis (GSEA) revealed significant enrichment in pathways such as proteasomal degradation, complement activation, and the integrin 3 pathway of the DEGs. Previous studies demonstrated that the proteasome system manages tumor suppressors and oncogenic proteins, and dysregulation of this system was common in various types of cancer [48]. Additionally, recent studies have suggested that complement activation promotes tumor progression in multiple ways [49]. These findings indicated that CRGs influence ESCC progression by regulating the tumor microenvironment and participating in the immune response.

In addition, we performed drug sensitivity analyses for seven drugs in the two risk groups, which included PLX4720, BMS.536924, BMS.754807, CGP.60474, NVP.TAE684, PF.02341066, and Sunitinib. Our results showed that the drug PLX4720 was more effective in the low-risk group of ESCC patients, whereas BMS.536924, BMS.754807, CGP.60474, NVP.TAE684, PF.02341066, and Sunitinib were more effective in the high-risk group of ESCC patients. PLX4720 is a selective inhibitor of BRAF V600E and specifically inhibits the MAPK/ERK signaling pathway [50]. BMS.536924 and BMS.754807 are IGF-1R inhibitors that activate the PI3K/AKT and MAPK signaling pathways by binding to their ligands, thereby promoting ESCA cell growth, proliferation, differentiation, and inhibiting apoptosis [51]. CGP.60474 is a CDK inhibitor that inhibits the cell cycle protein/CDK complex, which promotes cell cycle initiation and cell proliferation [51]. Activation of this cell growth cycle pathway is also prevalent in ESCA [50]. NVP.TAE684 is an ALK inhibitor that inhibits ALK rearrangement and the downstream related signaling pathways, resulting in tumor cell proliferation and survival [52]. PF.02341066 is an ATP-competitive multi-target protein kinase inhibitor that inhibits Met/ALK/ROS. Our findings may provide new therapeutic ideas for the targeted treatment of ESCC.

Furthermore, we observed that activated CD4 memory T cells, M0 macrophages, and tumor-associated macrophages were the most prevalent immune stromal cells in the tumor microenvironment. In addition, the expression level of PDHB was found to be significantly negatively correlated with the infiltration abundance of activated mast cells and naive CD4+ T cells in the low-risk group. In squamous cell carcinoma, the presence of activated mast cells has been correlated with disease progression, increased metastasis, and reduced patient survival [53]. CD4+ memory T cells inhibit tumor cell growth by promoting the proliferation of CD8+ memory T cells [54]. Moreover, mast cell inactivation was observed to suppress the accumulation of associated tumor-macrophages, resulting in reduced tumor cell proliferation, angiogenesis, and diminished tumor burden [55]. Therefore, our upcoming research will focus on further exploring the relationship between PDHB and these immune subsets. Taken together, we integrated 11 CRGs (ATP7B, CDKN2A, DLAT, DLD, FDX1, LIAS, LIPT1, MTF1, PDHA1, PDHB, and SLC31A1) to develop a nomogram for quantitative prediction. The findings demonstrated that the risk score model was capable of predicting the prognosis of ESCC patients, enhancing the clinical utility of this prognostic feature.

However, there are certain limitations that need to be addressed. Firstly, no further experimental validation has been conducted. In future studies, we intend to validate this prognostic model through *in vitro* experiments using cell lines and mouse models, and investigate the underlying mechanisms of this signature. Secondly, the reliability and stability of this prognostic model must be validated through prospective, multicenter studies and real-world data. These issues need to be addressed in future research.

## CONCLUSION

In conclusion, we have utilized an integrated bioinformatics approach to explore the expression and prognosis of CRGs in ESCC, which may serve as potential biomarkers for therapeutic selection. Moreover, we have developed a prognostic gene signature model and diagnostic model associated with ESCC. Additionally, our findings indicated that CRGs modulated ESCC initiation and progression by regulating the tumor microenvironment and immune response. Lastly, we have proposed a novel concept of ESCC targeted therapy. However, the underlying pathogenesis and molecular targets require further validation.

## Supplementary Materials

Supplementary Figure 1

Supplementary Tables
