# Supplementary material for: Identification and validation of a prognostic signature of cuproptosis-related genes for esophageal squamous cell carcinoma
Source: Aging (Albany NY). 2023 Sep 2;15(17):8993–9021. doi: 10.18632/aging.205012 (PMC10522377; doi:10.18632/aging.205012)
Supplement: Supplementary Figure 1 [file aging-15-205012-s001.pdf]

SUPPLEMENTARY FIGURE

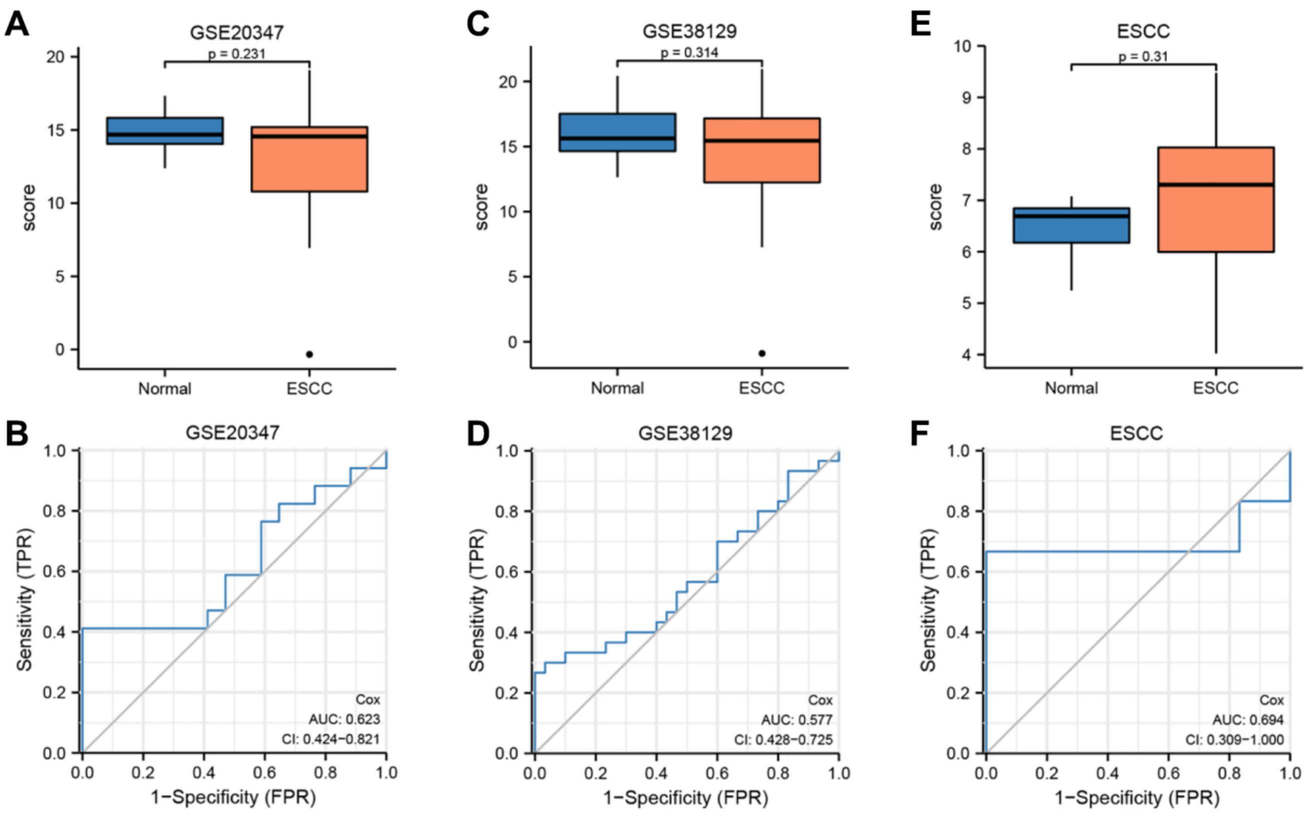

Supplementary Figure 1. The prognostic value of the LASSO-Cox regression prognosis risk model in three validation datasets (GSE2034, GSE38129, and ESCC). (**A**, **B**) Boxplots (**A**) and ROC curve (**B**) for the risk score levels in the ESCC and normal groups in GSE20347 dataset. (**C**, **D**) Boxplots (**C**) and ROC curve (**D**) for the risk score levels in the ESCC and normal groups in GSE38129 dataset. (**E**, **F**) Boxplots (**E**) and ROC curve (**F**) for the risk score levels in the ESCC and normal groups in ESCC dataset. $P \geq 0.05$ indicated no statistical significance; $P < 0.05$ was statistically significant. $P < 0.01$ was shown highly statistically significant. $P < 0.001$, which was extreme statistically significant. AUC value of ROC curves the closer to 1, the better diagnosis performance. AUC values more than 0.9 considered as having the capability to diagnose ESCC with excellent specificity and sensitivity, between 0.7 and 0.9 indicated specificity and sensitivity, less than 0.5 presented specificity and sensitivity. Abbreviations: LASSO: Least absolute shrinkage and selection operator; ESCC: esophageal squamous cell carcinoma; ROC: receiver operating characteristic curve.
