# Supplementary material for: Identification and validation of a prognostic signature of cuproptosis-related genes for esophageal squamous cell carcinoma
Source: Aging (Albany NY). 2023 Sep 2;15(17):8993–9021. doi: 10.18632/aging.205012 (PMC10522377; doi:10.18632/aging.205012)
Supplement: Supplementary Tables [file aging-15-205012-s002.pdf]

SUPPLEMENTARY TABLES

Supplementary Table 1. Clinical data for sequencing of patients with esophageal squamous cell carcinoma in our institution.

Characteristic	Levels	Overall
N		6
Gender, *n* (%)	Female	1 (16.7%)
	Male	5 (83.3)
Age, *n* (%)	≤60	4 (66.7%)
	>60	2 (33.3%)
Smoking, *n* (%)	Yes	4 (66.7%)
	No	2 (33.3%)
Pathologic T stage, *n* (%)	2	4 (66.7%)
	3	2 (33.3%)
Pathologic N stage, *n* (%)	0	4 (66.7%)
	2	2 (33.3%)
Pathologic TNM stage, *n* (%)	I	3 (50.0%)
	II	1 (16.7%)
	III	2 (33.3%)

Supplementary Table 2. The signature coefficients for each gene under different penalty coefficients in the LASSO regression.

Variable	Lambda.min
ATP7B	0.425630623
CDKN2A	−0.490982128
DLAT	−0.466209791
DLD	−0.314629868
FDX1	0.107707084
GLS	0
LIAS	−1.017948588
LIPT1	−0.899225738
MTF1	−0.23401496
PDHA1	0.821922748
PDHB	1.556412604
SLC31A1	0.16321755

Supplementary Table 3. Differentially expressed genes in TCGA-ESCC dataset high and low risk group.

Gene symbol					
ABCC2	CDKN2A	DCC	IGFN1	NXPE2	TFAP2B
ACTC1	CEND1	DNER	KCNU1	PAGE2	TFCP2L1
ACTL8	CH25H	DPT	KRT20	PCBP3	TFPI2
ADAM22	CHRNA9	DRD1	LCE1F	PLP1	TKTL1
ADAMTS18	CHST4	EGFR	MIOX	PRG4	TMEM200C
ALKAL2	CILP	EZHIP	MS4A1	PWWP3B	TMEM202
BPIFB2	CKM	FRMD1	MT1A	RORB	TRIM71
C10orf90	CPLX2	GFRA3	MUC6	RXFP1	UPK1A
C11orf88	CRYBA2	GOLGA6L7	NOBOX	SFTPA2	VWA2
C1QL1	CXCL12	GRB14	NPTX1	SLC8A2	ZFP57

C9orf24	DCAF4L2	HBE1	NR5A1	TBX18	ZNF541
CDH12	DCAF8L2	IGFBP1	NTN3	TEPP	

Supplementary Table 4. GO enrichment analysis results of differentially expressed genes.

Ontology	ID	Description	GeneRatio	BgRatio	*p* value	*p*.adjust	*q* value
BP	GO: 0008344	adult locomotory behavior	4/60	77/18670	1.09e-04	0.099	0.089
BP	GO: 0001764	neuron migration	5/60	157/18670	1.48e-04	0.099	0.089
CC	GO: 0099055	integral component of postsynaptic membrane	4/66	117/19717	6.39e-04	0.045	0.040
CC	GO: 0098936	intrinsic component of postsynaptic membrane	4/66	122/19717	7.48e-04	0.045	0.040
CC	GO: 0099699	integral component of synaptic membrane	4/66	152/19717	0.002	0.045	0.040
CC	GO: 0099060	integral component of postsynaptic specialization membrane	3/66	74/19717	0.002	0.045	0.040
CC	GO: 0098948	intrinsic component of postsynaptic specialization membrane	3/66	77/19717	0.002	0.045	0.040
CC	GO: 0099240	intrinsic component of synaptic membrane	4/66	164/19717	0.002	0.045	0.040
CC	GO: 0062023	collagen-containing extracellular matrix	6/66	406/19717	0.002	0.045	0.040
CC	GO: 0099634	postsynaptic specialization membrane	3/66	101/19717	0.005	0.079	0.070
CC	GO: 0098978	glutamatergic synapse	5/66	349/19717	0.006	0.092	0.082
MF	GO: 0005201	extracellular matrix structural constituent	5/62	163/17697	2.64e-04	0.051	0.049

Supplementary Table 5. GSEA analysis of TCGA-ESCC dataset.

Description	setSize	enrichmentScore	NES	*p* value	*q* values
REACTOME_SMOOTH_MUSCLE_CONTRACTION	38	0.67108	2.08286	0.00215	0.15306
WP_MIRNA_TARGETS_IN_ECM_AND_MEMBRANE_RECEPTORS	22	0.74701	2.06312	0.00227	0.15306
WP_STRIATED_MUSCLE_CONTRACTION_PATHWAY	38	0.65059	2.01924	0.00215	0.15306
WP_ELECTRON_TRANSPORT_CHAIN_OXPHOS_SYSTEM_IN_MITOCHONDRIA	90	0.55785	2.00529	0.00205	0.15306
WP_MYOMETRIAL_RELAXATION_AND_CONTRACTION_PATHWAYS	156	0.52354	1.999	0.00232	0.15306
REACTOME_INITIAL_TRIGGERING_OF_COMPLEMENT	22	0.72334	1.99774	0.00227	0.15306
BIOCARTA_COMP_PATHWAY	19	0.75325	1.9961	0.00442	0.1952
NABA_PROTEOGLYCANS	35	0.65393	1.992	0.0022	0.15306
KEGG_VASCULAR_SMOOTH_MUSCLE_CONTRACTION	114	0.53649	1.98928	0.00219	0.15306
REACTOME_ACYL_CHAIN_REMODELLING_OF_PG	18	0.7545	1.96668	0.00454	0.1952

WP_PROTEASOME_DEGRADATION	62	0.5837	1.96244	0.00218	0.15306
BIOCARTA_CLASSIC_PATHWAY	14	0.80088	1.94794	0.00458	0.1952
REACTOME_BINDING_AND_UPTAKE_OF_LIGANDS_BY_SCAVENGER_RECEPTORS	42	0.61753	1.93018	0.00218	0.15306
REACTOME_MUSCLE_CONTRACTION	205	0.48585	1.92507	0.00221	0.15306
KEGG_DILATED_CARDIOMYOPATHY	90	0.53424	1.92044	0.00205	0.15306
REACTOME_STRIATED_MUSCLE_CONTRACTION	36	0.62449	1.91763	0.00222	0.15306
WP_COMPLEMENT_ACTIVATION	22	0.69281	1.91341	0.00227	0.15306
REACTOME_ACYL_CHAIN_REMODELLING_OF_PI	17	0.7468	1.90725	0.00454	0.1952
REACTOME_SCAVENGING_BY_CLASS_A_RECEPTORS	19	0.71518	1.8952	0.00442	0.1952
PID_INTEGRIN3_PATHWAY	43	0.59355	1.864	0.0022	0.15306

Supplementary Table 6. Patient characteristics of ESCC patients in the TCGA datasets.

Characteristic	Levels	Overall
n		80
T stage, *n* (%)	T1	8 (10.1%)
	T2	27 (34.2%)
	T3	41 (51.9%)
	T4	3 (3.8%)
N stage, *n* (%)	N0	46 (59%)
	N1	26 (33.3%)
	N2	5 (6.4%)
	N3	1 (1.3%)
M stage, *n* (%)	M0	70 (95.9%)
	M1	3 (4.1%)
Pathologic stage, *n* (%)	Stage I	7 (8.9%)
	Stage II	47 (59.5%)
	Stage III	22 (27.8%)
	Stage IV	3 (3.8%)
Age, *n* (%)	≤60	51 (63.7%)
	>60	29 (36.2%)
Gender, *n* (%)	Female	12 (15%)
	Male	68 (85%)
